# Discovery and Activity Evaluation of Novel Dibenzoxazepinone Derivatives as Glycogen Phosphorylase Inhibitors

**DOI:** 10.3390/molecules30244797

**Published:** 2025-12-16

**Authors:** Dongrui Liu, Zhiwei Yan, Youde Wang, Shuai Li, Yachun Guo, Tienan Wang, Jinjia Guo, Liying Zhang

**Affiliations:** 1 Laboratory of Traditional Chinese Medicine Research and Development of Hebei Province, Institute of Traditional Chinese Medicine, Chengde Medical University, Chengde 067000, China; 17332623448@163.com (D.L.); cdyanzhiwei@163.com (Z.Y.); wangyoude8686@126.com (Y.W.); cmuyhls@163.com (S.L.); cdwangtienan@163.com (T.W.); 2 Department of Pathogen Biology, Chengde Medical University, Chengde 067000, China; gyc123263295@126.com; 3 Jingfukang Pharmaceutical Group Co., Ltd., Chengde 067000, China; 13932405279@139.com

**Keywords:** diabetes, glycogen phosphorylase inhibitors, dibenzoxazepinone derivatives, hypoglycemic, glycogenolysis

## Abstract

Inhibition of glycogen phosphorylases (GP) has been regarded as a therapeutic strategy for blood glucose control in diabetes. In this study, a series of novel dibenzoxazepinone derivatives was synthesized. The in vitro activity screening results indicated that compound **Id** most significantly inhibited glycogen phosphorylase (GP) activity, with an IC_50_ of 266 ± 1 nM, which was superior to the positive control drug **PSN-357**, a Phase II clinical GP inhibitor from Japan’s OSI Corporation. In vivo experiments showed that **Id** could significantly reduce blood glucose levels in adrenaline-induced acute hyperglycemic mice and high-fat-diet-induced obese and diabetic (DIO) mice.

## 1. Introduction

Type 2 diabetes mellitus (T2DM) is a metabolic disorder primarily characterized by persistent hyperglycemia. According to data from the International Diabetes Federation (IDF) in 2021, over 500 million people worldwide are affected by diabetes, with more than 90% having T2DM [1]. This number is projected to rise to 1.31 billion by 2050 [2].

Persistent hyperglycemia is closely associated with increased hepatic glucose output, with glycogenolysis being one of the primary sources of hepatic glucose output [3,4]. Glycogen phosphorylase (GP), a key rate-limiting enzyme in the glycogenolysis pathway, significantly enhances hepatic glucose output when its activity is overactivated, thereby exacerbating hyperglycemia [5]. Therefore, inhibition of GP has emerged as a potential strategy for the treatment of T2DM [6,7,8].

**Figure 1:**
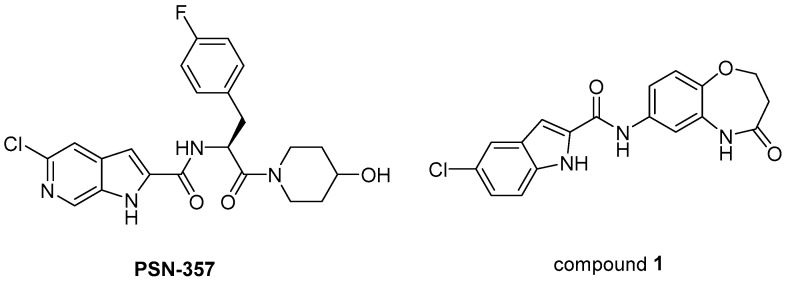
Structure of **PSN-357** and compound **1**.

Currently, various types of GP inhibitors with different structural classes have been reported. These include the anthocyanins and flavonoid derivatives that target quercetin site [9,10,11,12], glucosyl inhibitors that act on catalytic sites [13,14,15], benzimidazole-based compounds [16,17,18], and indole scaffold allosteric inhibitors that bind to the novel allosteric site [19,20,21]. All of these compounds have shown promising inhibitory activities in in vitro experiments. Some GP inhibitors, such as **PSN-357** (Figure 1) from OSI Pharmaceuticals and **CP320626** from Pfizer, have even advanced to Phase II clinical trials [22].

In our previous research, we identified a series of benzazepinone compounds with excellent GP inhibitory activity [23], with the representative compound **1** displaying an inhibition activity of 0.25 ± 0.05 μM. However, the structure–activity relationship (SAR) of this class of compounds remains poorly understood. To address this, the present study designs and synthesizes two novel series of dibenzooxazepinone derivatives as structural analog comparative scaffolds, building upon our previous findings. These derivatives serve to probe the core pharmacophore and facilitate a deeper understanding of the SAR of benzazepinone-based GP inhibitors, providing valuable insights into the influence of structural modifications on biological activity.

## 2. Results and Discussion

### 2.1. Chemistry

**Scheme 1:**
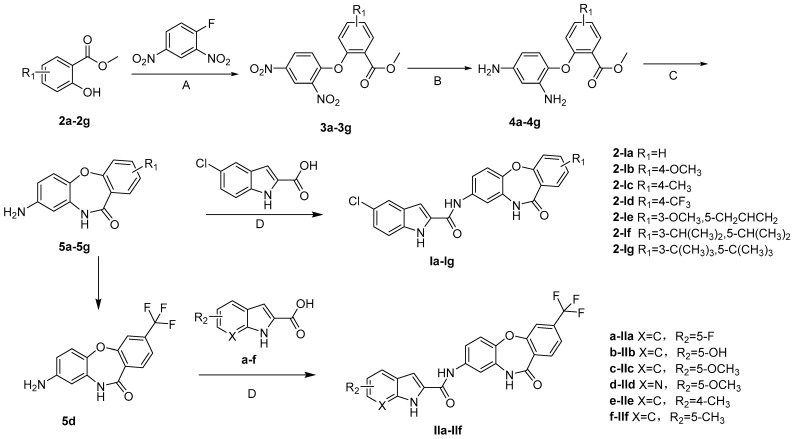
Synthesis routes of **Ia**–**Ig** and **IIa**–**IIf**. Reagents and conditions: (A) K_2_CO_3_ (1.4 eq), r.t., acetone, 54 h, [67.5–89.2%]; (B) HCOONH_4_ (8 eq), Pd/C (0.1 eq), ethanol, r.t., 1 h, [65–96%]; (C) THF/H_2_O, HCl, reflux, under N_2_, overnight, [25.8–92%]; (D) DMF, HATU (1.5 eq), Et_3_N (4.6 eq), 45 °C, overnight, [26.5–72%].

As shown in Scheme 1, various methyl salicylate derivatives **2a**–**2g** were obtained via procedures similar to those reported previously [24]. Subsequently, **2a**–**2g** were combined with 1-fluoro-2,4-dinitrobenzene via a substitution reaction, yielding nitro compounds **3a**–**3g**. Then, reduction of the nitro groups using palladium on carbon (Pd/C) and ammonium formate yielded the amine intermediates **4a**–**4g**. Cyclization of **4a**–**4g** was achieved under reflux in tetrahydrofuran/water (THF/H_2_O) with hydrochloric acid under nitrogen, constructing the key dibenzooxazepinone core **5a**–**5g**. Finally, coupling of **5a**–**5g** with 5-chloroindole-2-carboxylic acid in *N*,*N*-dimethylformamide (DMF) at 45 °C, using 2-(7-azabenzotriazol-1-yl)-*N*,*N*,*N*′,*N*′-tetramethyluronium hexafluorophosphate (HATU) and triethylamine (Et_3_N) as coupling agents, delivered the target compounds **Ia**–**Ig**.

To explore structural diversity, a second series was prepared from intermediate **5d**. Coupling of **5d** with various substituted indole-2-carboxylic acids (**a**–**f**) under identical conditions (HATU, Et_3_N, DMF, 45 °C) provided the final compounds **IIa**–**IIf**.

### 2.2. Phosphorylase Enzyme Assay and SAR Analysis

The synthesized compounds were evaluated in an enzyme inhibition assay against rabbit muscle glycogen phosphorylase a (RMGPa), a commercially available enzyme that shares considerable sequence similarity with human liver GPa [20]. As described previously, the activity of RMGPa was measured by detecting the release of phosphate from glucose-1-phosphate in the direction of glycogen synthesis. **PSN-357**, a GP inhibitor in clinical Phase II, was used as positive control.

As shown in Table 1, most of the dibenzooxazepinone derivatives (**Ia**–**Ig**, **IIa**–**IIf**) exhibit moderate to good inhibitory potency against RMGPa. Among them, compound **Id** displayed the highest inhibitory activity with an IC_50_ value of 266 ± 1 nM. This potency was not only superior to that of the positive control **PSN-357** (IC_50_ = 420 ± 10 nM) but also comparable to the previously reported parent compound **1** (IC_50_ = 250 ± 50 nM). Moreover, **Ib** and **IIf** were still more active than the positive control **PSN-357**, although they had slightly weaker activity compared to derivative **Id**.

Preliminary analysis suggests that the electronic properties and steric volume of substituents on the azepinone ring significantly affect the inhibitory activity. Compound **Id**, featuring a strong electron-withdrawing group (EWG), exhibits optimal activity. In contrast, electron-donating groups (EDGs) generally impair activity, as seen in compound **Ic**, which shows no activity. Notably, compound **Ib**, despite bearing an EDG, demonstrates significant activity, likely due to the methoxy group acting as a hydrogen bond acceptor. Additionally, introducing bulky substituents (**Ie**, **If**, **Ig**) results in a sharp decrease or loss of activity, indicating that steric hindrance is a key factor.

In general, modification of the indole ring at 5-position results in decreased GPa inhibitory activity (**IIb** vs. **IIc** vs. **IIe**). This trend in activity may be due to the decreasing polarity from the –OH group to the -OCH_3_ and -CH_3_ groups. However, the size of the substituent in the indole ring is also important. The inhibitory activity increased with the size of the halogen atom, as evidenced by compound **Id** (Cl) being more potent than compound **IIa** (F). Compared to compound **Id**, replacing the aromatic carbon atom with a nitrogen atom would significantly reduce its inhibitory activity, indicating that the carbon skeleton of the indole ring is crucial (**IId** vs. **Id**).

In conclusion, this study confirms that the azepinone core and aromatic rings are important for GPa inhibition. The preliminary SAR reveals that the activity is finely tuned by a complex interplay of electronic, steric, and polar effects, rather than by a single predictable parameter. Furthermore, the selectivity of compound **Id** for other GP isoforms and its potential off-target effects remain to be elucidated through systematic follow-up studies.

### 2.3. In Vivo Studies

The bioassay data revealed that compound **Id** exhibited excellent in vitro activity against RMGPa with the lowest IC_50_ of 266 ± 1 nM. Based on this observation, a systematic investigation of compound **Id** about its antihyperglycemic effect was carried out.

**Figure 2:**
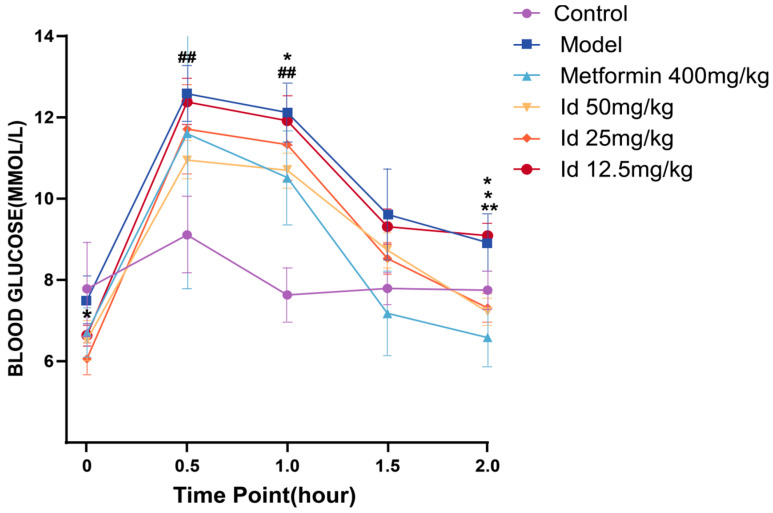
Time course of the glucose-lowering effect of oral administration of compound **Id** in hyperglycemic mice induced by adrenaline (n = 10). # *p* < 0.05, ## *p* < 0.01 vs. Control group; * *p* < 0.05, ** *p* < 0.01 vs. Model group.

First, its ability was investigated by oral administration with one dose of 0.5% CMC-NA (model group) or compound **Id** at different concentrations (12.5, 25 and 50 mg/kg) for treatment. Metformin (400 mg/kg) was selected as a positive control. As shown in Figure 2, compared with the model group, both the moderate- and high-dose groups exhibited a significant decline in blood glucose after approximately 2 h following oral administration (7.30 ± 0.35 and 7.20 ± 0.34 vs. 8.91 ± 0.69 mmol/L, *p* < 0.05). However, this hypoglycemic effect was slightly less potent than that of the metformin group (6.57 ± 0.71 mmol/L vs. 8.91 ± 0.69 mmol/L, *p* < 0.01). In contrast, no hypoglycemic effect was observed in the low-dose group of compound **Id**.

**Figure 3:**
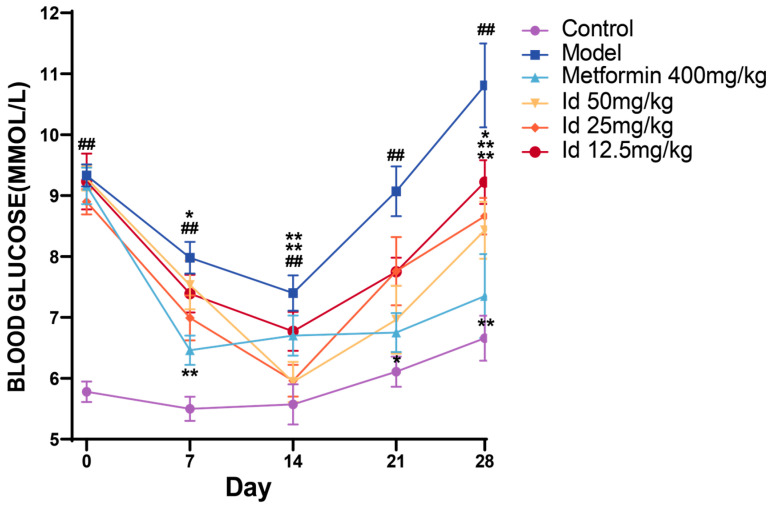
Time course of hypoglycemic effect of compound **Id** after oral administration in high-fat-diet-induced diabetic DIO mice (n = 10). # *p* < 0.05, ## *p* < 0.01 vs. Control group; * *p* < 0.05, ** *p* < 0.01 vs. Model group.

Subsequently, in accordance with the previously reported protocol [25], the long-term hypoglycemic effect of compound **Id** was evaluated by using high-fat-diet-induced diabetic mice (DIO). The low-dose (12.5 mg/kg), moderate-dose (25 mg/kg) and high-dose (50 mg/kg) groups of mice on the high-fat diet were treated by oral gavage once every day for 4 weeks. Metformin was still serving as the positive control. As presented in Figure 3, during the long-term administration, the high-dose group showed a significant hypoglycemic effect on day 7 (7.53 ± 0.40 mmol/L vs. 7.98 ± 0.26 mmol/L, *p* < 0.05). By day 14, both the moderate- and high-dose groups of mice displayed remarkable blood-glucose-lowering effects (5.96 ± 0.26 mmol/L and 5.94 ± 0.33 mmol/L vs. 7.40 ± 0.29 mmol/L, *p* < 0.01). Finally, on day 28, all dose groups exhibited statistically significant hypoglycemic activity. Among them, the moderate- and high-dose groups exerted an apparent effect (8.43 ± 0.47 and 8.66 ± 0.30 mmol/L vs. 10.81 ± 0.69 mmol/L, *p* < 0.01). The low-dose group exhibited a similar glucose-lowering action to the other groups (9.22 ± 0.36 mmol/L, *p* < 0.05).

In this study, the potent antihyperglycemic effect of compound **Id** is primarily attributed to its direct inhibition of glycogen phosphorylase (GP), as demonstrated by its nanomolar activity against purified RMGPa and its rapid efficacy in the adrenaline-induced model targeting the glycogenolytic pathway. While our present data do not entirely rule out potential effects on other glucose metabolism pathways or intestinal absorption, the consistent evidence from both in vitro and in vivo models strongly suggests that GP inhibition is the dominant mechanism. Further investigations are planned to explore potential off-target effects.

## 3. Materials and Methods

### 3.1. Materials and General Methods

All commercially available solvents and reagents were purchased from J&K Chemical Co., Ltd. (Beijing, China) and WuXi AppTec (Wuxi, China). The melting points (M.p.) of the compounds were determined using an RY-1 melting point apparatus(Tianjin Tianfen Analytical Instrument Factory, Tianjin, China). Nuclear Magnetic Resonance (NMR) experiments were performed on a Bruker Avance III 400 MHz spectrometer and a Bruker Fourier 300 MHz spectrometer (Bruker Corporation, Karlsruhe, Germany), with the spectra internally referenced to the residual solvent signals of tetramethylsilane (TMS, δ = 0.00 ppm). Positive and negative ion LC-MS data were acquired at 303 K using an Agilent LC/MSD 1200 Series quadrupole mass spectrometer, which was equipped with a 50 × 4.6 mm (5 µm) ODS column (Agilent Technologies, Santa Clara, CA, USA). High-Resolution Mass Spectrometry (HRMS) analyses were conducted on an Agilent 6540 QTOF mass spectrometer (Agilent Technologies, Santa Clara, CA, USA). All final compounds were purified, and their purity was verified by High-Performance Liquid Chromatography (HPLC). The HPLC analysis was carried out on an Agilent 1260 system (Agilent Technologies, Singapore) fitted with an Agilent Poroshell 120 EC-C18 column (4.6 mm × 150 mm, 4.0 µm). Reversed-phase preparative HPLC (prep-HPLC) experiments were performed via flash chromatography using a Welchrom C18 column (150 × 20 mm, Agela Technologies, Tianjin, China). Reaction progress for all compounds was monitored by thin-layer chromatography (TLC). The majority of compounds achieved a purity of >95%; the chromatographic purity data for representative compounds are provided in the Supplementary Information.

### 3.2. General Procedure for Compound Synthesis

#### 3.2.1. General Procedure for the Synthesis of Compounds **2a**, **2d**, **2f**, **2g**

Methyl 2-hydroxybenzoate (**2a**)

Salicylic acid (5 g, 36.2 mmol) was dissolved in methanol (60 mL). Concentrated sulfuric acid (4 mL) was added slowly to the above solution, and the reaction mixture was refluxed at 80 °C for 72 h with stirring. After the reaction was completed, the reaction mixture was cooled to room temperature, and the solvent was evaporated to dryness under reduced pressure. The resulting residue was dissolved in ethyl acetate (EA) (60 mL), and the organic phase was washed with saturated brine (15 mL × 3). The washed organic phase was dried over anhydrous magnesium sulfate (MgSO_4_) for 2–3 h. Subsequently, the crude product was purified by silica gel column chromatography using petroleum ether (PE) as the eluent to obtain a colorless liquid (3.8 g, 69% yield). HPLC analysis: 100.0%. m.p. None. ESI-MS *m*/*z*: 153.5 (M + H)^+^. ^1^H-NMR (400 MHz, CDCl_3_): 3.97 (s, 3H), 6.90 (t, *J* = 7.2 Hz, 1H), 7.00 (d, *J* = 8.0 Hz, 1H), 7.48 (t, *J* = 8.0 Hz, 1H), 7.85 (d, *J* = 8.0 Hz, 1H), 10.78 (s, 1H). ^13^C-NMR (100 MHz, CDCl_3_): 170.6, 161.6, 135.7, 129.9, 119.2, 117.6, 112.4, 52.3.

Methyl 2-hydroxy-4-(trifluoromethyl)benzoate (**2d**)

Compound **2d** was prepared from 4-trifluoromethylsalicylic acid according to the procedure described in **2a** as a colorless liquid (0.9 g, 42%). HPLC analysis: 100.0%. m.p. None. ESI-MS *m*/*z*: 219.0 (M-H)^−^. ^1^H-NMR (400 MHz, CDCl_3_): 4.01 (s, 3H), 7.14 (dd, *J* = 8.4, 1.2 Hz, 1H), 7.27 (s, 1H), 7.97 (d, *J* = 8.4 Hz, 1H), 10.91 (s, 1H). ^13^C-NMR (100 MHz, CDCl_3_): 169.7, 161.5, 136.9 (q, *J* = 32.7 Hz), 130.8, 124.5, 121.8, 115.5 (q, *J* = 3.6 Hz), 115.0 (q, *J* = 3.9 Hz), 52.8.

Methyl 2-hydroxy-3,5-diisopropylbenzoate (**2f**)

Compound **2f** was prepared from 3,5-diisopropylsalicylic acid according to the procedure described in **2a** as a colorless liquid (1.85 g, 58%). HPLC analysis: 100.0%. m.p. None. ESI-MS *m*/*z*: None. ^1^H-NMR (400 MHz, CDCl_3_): 1.24 (t, *J* = 6.4 Hz, 12H), 2.79–2.90 (m, 1H), 3.31–3.39 (m, 1H), 3.93 (s, 3H), 7.26 (d, *J* = 2.4 Hz, 1H), 7.53 (d, *J* = 2.4 Hz, 1H), 10.96 (s, 1H). ^13^C-NMR (100 MHz, CDCl_3_): 171.3, 157.5, 138.9, 136.6, 130.9, 124.1, 111.3, 52.1, 33.5, 26.8, 24.1, 22.4.

Methyl 3,5-di-tert-butyl-2-hydroxybenzoate (**2g**)

Compound **2g** was prepared from 3,5-di-tert-butyl salicylic acid according to the procedure described in **2a** as a white solid (1.39 g, 44%). HPLC analysis: 100.0%. m.p. 72–74 °C. ESI-MS *m*/*z*: 287.4 (M + Na)^+^. ^1^H-NMR (400 MHz, CDCl_3_): 1.30 (s, 9H), 1.43 (s, 9H), 3.93 (s, 3H), 7.52 (d, *J* = 2.4 Hz, 1H), 7.71 (d, *J* = 2.4 Hz, 1H), 11.35 (s, 1H). ^13^C-NMR (100 MHz, CDCl_3_): 171.8, 159.0, 140.4, 137.2, 130.4, 123.6, 111.3, 52.2, 35.2, 34.3, 31.4, 29.4.

#### 3.2.2. General Procedure for the Synthesis of Compounds **3a**–**3g**

2,4-Dinitrofluorobenzene (1.0 eq) was dissolved in acetone (20 mL). With continuous stirring, anhydrous potassium carbonate (K_2_CO_3_) (1.4 eq) and methyl salicylate derivatives **2a**–**2g** (1.4 eq) were added to the above solution. The reaction mixture was stirred at room temperature for 54 h. After cooling at room temperature, the mixture was diluted with an appropriate amount of distilled water and then extracted with EA (15 mL × 3). The combined organic phases were washed with saturated brine (15 mL × 3), dried over anhydrous Na_2_SO_4_, and evaporated. The residue was purified by silica gel chromatography with PE/EA (15:1) to obtain compounds **3a**–**3g**.

Methyl 2-(2,4-dinitrophenoxy)benzoate (**3a**)

White solids (2.1 g, 88%). HPLC analysis: 100.0%. m.p. 87–89 °C. ESI-MS *m*/*z*: None. ^1^H-NMR (400 MHz, CDCl_3_): 3.76 (s, 3H), 6.83 (d, *J* = 9.2 Hz, 1H), 7.29 (d, *J* = 6.0 Hz, 1H), 7.48 (t, *J* = 7.6 Hz, 1H), 7.72 (t, *J* = 7.6 Hz, 1H), 8.12 (d, *J* = 7.6 Hz, 1H), 8.28 (d, *J* = 9.2 Hz, 1H), 8.89 (s, 1H). ^13^C-NMR (100 MHz, CDCl_3_): 164.3, 156.8, 152.3, 141.1, 138.8, 134.8, 133.0, 128.7, 127.2, 123.6, 123.5, 122.1, 117.1, 52.5.

Methyl 2-(2,4-dinitrophenoxy)-4-methoxybenzoate (**3b**)

White solids (1.5 g, 86%). HPLC analysis: 100.0%. m.p. 130–133 °C. ESI-MS *m*/*z*: None. ^1^H-NMR (400 MHz, CDCl_3_): 3.69 (s, 3H), 3.90 (s, 3H), 6.77 (d, *J* = 2.8 Hz, 1H), 6.84 (d, *J* = 9.2 Hz, 1H), 6.95 (dd, *J* = 9.2, 2.8 Hz, 1H), 8.08 (d, *J* = 8.4 Hz, 1H), 8.27 (dd, *J* = 9.2, 2.8 Hz, 1H), 8.88 (d, *J* = 2.8 Hz, 1H). ^13^C-NMR (100 MHz, CDCl_3_): 164.7, 163.9, 156.8, 154.0, 141.1, 138.7, 134.6, 128.7, 122.1, 116.9, 115.2, 112.8, 109.9, 56.0, 52.2.

Methyl 2-(2,4-dinitrophenoxy)-4-methylbenzoate (**3c**)

Pale yellow solid (1.5 g, 84.3%). HPLC analysis: 97.4%. m.p. 117–119 °C. ESI-MS *m*/*z*: None. ^1^H-NMR (400 MHz, CDCl_3_): 2.48 (s, 3H), 3.73 (s, 3H), 6.83 (d, *J* = 9.2 Hz, 1H), 7.10 (s, 1H), 7.28 (s, 1H), 8.01 (d, *J* = 8.0 Hz, 1H), 8.28 (dd, *J* = 9.2, 2.8 Hz, 1H), 8.89 (d, *J* = 2.8 Hz, 1H). ^13^C-NMR (100 MHz, CDCl_3_): 164.2, 156.9, 152.3, 146.5, 141.0, 138.7, 132.9, 128.7, 128.0, 124.0, 122.1, 120.4, 117.1, 52.3, 21.5.

Methyl 2-(2,4-dinitrophenoxy)-4-(trifluoromethyl)benzoate (**3d**)

Pale yellow solid (650 mg, 67.5%). HPLC analysis: 100.0%. m.p. 91–93 °C. ESI-MS *m*/*z*: None. ^1^H-NMR (400 MHz, CDCl_3_): 3.81 (s, 3H), 6.85 (d, *J* = 9.2 Hz, 1H), 7.56 (s, 1H), 7.74 (d, *J* = 8.0 Hz, 1H), 8.24 (d, *J* = 8.4 Hz, 1H), 8.35 (dd, *J* = 9.2, 2.8 Hz, 1H), 8.92 (d, *J* = 2.8 Hz, 1H). ^13^C-NMR (100 MHz, CDCl_3_): 163.3, 155.8, 152.5, 141.8, 139.1, 136.5 (q, *J* = 33.7 Hz), 133.8, 128.9, 127.0, 123.8 (q, *J* = 3.7 Hz), 122.9 (d, *J* = 271.7 Hz), 122.2, 120.6 (q, *J* = 3.7 Hz), 117.3, 122.3, 118.1, 53.0.

Methyl 5-allyl-2-(2,4-dinitrophenoxy)-3-methoxybenzoate (**3e**)

Pale yellow solid (1.7 g, 87.6%). HPLC analysis: 100.0%. m.p. 129–132 °C. ESI-MS *m*/*z*: None. ^1^H-NMR (400 MHz, CDCl_3_): 3.46 (d, *J* = 6.8 Hz, 2H), 3.76 (s, 3H), 3.80 (s, 3H), 5.15 (d, *J* = 7.2 Hz, 1H), 5.19 (s, 1H), 5.93–6.03 (m, 1H), 6.80 (d, *J* = 9.2 Hz, 1H), 7.05 (d, *J* = 1.6 Hz, 1H), 7.42 (d, *J* = 1.6 Hz, 1H), 8.26 (dd, *J* = 9.2, 2.8 Hz, 1H), 8.88 (d, *J* = 2.8 Hz, 1H). ^13^C-NMR (100 MHz, CDCl_3_): 164.7, 156.6, 152.0, 141.0, 140.0, 139.0, 138.3, 135.9, 128.6, 125.0, 123.1, 122.1, 117.3, 117.1, 116.9, 56.4, 52.5, 39.9.

Methyl 2-(2,4-dinitrophenoxy)-3,5-diisopropylbenzoate (**3f**)

White solid (1.4 g, 68.6%). HPLC analysis: 96.4%. m.p. 121–124 °C. ESI-MS *m*/*z*: None. ^1^H-NMR (400 MHz, CDCl_3_): 1.22 (dd, *J* = 20.8, 6.0 Hz, 6H), 1.31 (d, *J* = 6.8 Hz, 6H), 2.97–3.09 (m, 2H), 3.69 (s, 3H), 6.71 (d, *J* = 9.2 Hz, 1H), 7.47 (d, *J* = 2.0 Hz, 1H), 7.76 (d, *J* = 2.0 Hz, 1H), 8.25 (dd, *J* = 9.2, 2.8 Hz, 1H), 8.88 (d, *J* = 2.8 Hz, 1H). ^13^C-NMR (100 MHz, CDCl_3_): 164.9, 157.2, 148.0, 146.9, 142.4, 140.8, 138.2, 130.3, 128.6, 128.1, 123.1, 122.1, 116.1, 52.4, 33.9, 27.3, 23.9, 22.3.

Methyl 3,5-di-tert-butyl-2-(2,4-dinitrophenoxy)benzoate (**3g**)

Grayish-white solid (1.45 g, 89.2%). HPLC analysis: 97.1%. m.p. 192–195 °C. ESI-MS *m*/*z*: None. ^1^H-NMR (400 MHz, CDCl_3_): 1.38 (s, 9H), 1.40 (s, 9H), 3.59 (s, 3H), 6.70 (d, *J* = 9.2 Hz, 1H), 7.75 (d, *J* = 2.4 Hz, 1H), 7.88 (d, *J* = 2.4 Hz, 1H), 8.23 (dd, *J* = 9.2, 2.8 Hz, 1H), 8.86 (d, *J* = 2.8 Hz, 1H). ^13^C-NMR (100 MHz, CDCl_3_): 165.3, 157.6, 149.3, 148.8, 143.3, 140.8, 139.0, 129.8, 128.5, 127.9, 122.8, 121.9, 116.6, 52.3, 35.6, 35.0, 31.3, 30.6.

#### 3.2.3. General Procedure for the Synthesis of Compounds **4a**–**4g**

HCOONH_4_ (8 eq) and Pd/C (0.1 eq) were added to a solution of **3a**–**3g** (1 eq) in anhydrous ethanol (20 mL). The reaction mixture was stirred at r.t. for 1 h. Then the insoluble solid was removed by filtration. The filtrate was concentrated in a vacuum, and then the given residue was purified by silica gel chromatography with PE/EA (1/1) to obtain compounds **4a**–**4g**.

Methyl 2-(2,4-diaminophenoxy)benzoate (**4a**)

Gray solid (634 mg, 65%). HPLC analysis: 97.5%. m.p. 129–132 °C. ESI-MS *m*/*z*: None. ESI-MS *m*/*z*: 258.9 (M + H)^+^. ^1^H-NMR (400 MHz, CDCl_3_): 3.77 (s, 4H), 3.93 (s, 3H), 6.09 (dd, *J* = 8.4, 2.8 Hz, 1H), 6.18 (d, *J* = 2.4 Hz, 1H), 6.79 (d, *J* = 8.4 Hz, 1H), 6.91 (d, *J* = 8.4 Hz, 1H), 7.05 (td, *J* = 7.6, 0.8 Hz, 1H), 7.37 (td, *J* = 8.0, 2.0 Hz, 1H), 7.81 (dd, *J* = 8.0, 1.6 Hz, 1H). ^13^C-NMR (100 MHz, CDCl_3_): 167.0, 158.0, 144.3, 140.0, 134.9, 133.4, 131.3, 122.7, 121.6, 120.8, 115.9, 105.3, 103.2, 52.2.

Methyl 2-(2,4-diaminophenoxy)-4-methoxybenzoate (**4b**)

Gray oily (0.65 g, 75.5%). HPLC analysis: 55.4%. m.p. None. ESI-MS *m*/*z*: 289.0 (M + H)^+^. ^1^H-NMR (400 MHz, CDCl_3_): 3.69 (s, 4H), 3.71 (s, 3H), 3.87 (s, 3H), 6.07 (dd, *J* = 8.4, 2.4 Hz, 1H), 6.15 (d, *J* = 2.4 Hz, 1H), 6.41 (d, *J* = 2.0 Hz, 1H), 6.55 (dd, *J* = 8.8, 2.0 Hz, 1H), 6.77 (d, *J* = 8.4 Hz, 1H), 7.83 (d, *J* = 8.4 Hz, 1H). ^13^C-NMR (100 MHz, CDCl_3_): 166.5, 164.1, 160.3, 144.3, 140.0, 134.7, 133.4, 122.7, 112.8, 107.1, 105.4, 103.3, 101.8, 55.4, 51.9.

Methyl 2-(2,4-diaminophenoxy)-4-methylbenzoate (**4c**)

Brown liquid (1 g, 91%). HPLC analysis: 87.1%. m.p. None. ESI-MS *m*/*z*: 272.9 (M + H)^+^. ^1^H-NMR (400 MHz, CDCl_3_): 2.25 (s, 3H), 3.77 (s, 4H), 3.89 (s, 3H), 6.09 (dd, *J* = 8.4, 2.0 Hz, 1H), 6.17 (d, *J* = 1.6 Hz, 1H), 6.68 (s, 1H), 6.77 (d, *J* = 8.4 Hz, 1H), 6.83 (d, *J* = 8.0 Hz, 1H), 7.71 (d, *J* = 7.6 Hz, 1H). ^13^C-NMR (100 MHz, CDCl_3_): 167.0, 158.2, 144.6, 144.1, 140.0, 135.0, 131.5, 122.8, 122.6, 117.7, 116.5, 105.4, 103.3, 52.1, 21.8.

Methyl 2-(2,4-diaminophenoxy)-4-(trifluoromethyl)benzoate (**4d**)

Yellow solid (353 mg, 88.7%). HPLC analysis: 92.7%. m.p. 78–81 °C. ESI-MS *m*/*z*: 326.9 (M + H)^+^. ^1^H-NMR (400 MHz, CDCl_3_): 3.73 (br s, 4H), 3.96 (s, 3H), 6.13 (dd, *J* = 8.4, 2.8 Hz, 1H), 6.20 (d, *J* = 2.8 Hz, 1H), 6.81 (d, *J* = 7.6 Hz, 1H), 7.14 (s, 1H), 7.30 (d, *J* = 8.4 Hz, 1H), 7.89 (d, *J* = 8.0 Hz, 1H). ^13^C-NMR (100 MHz, CDCl_3_): 166.1, 158.1, 144.7, 139.9, 134.9 (q, *J* = 32.8 Hz), 134.0, 131.8, 124.0, 123.2 (d, *J* = 271.3 Hz), 122.7, 118.2 (q, *J* = 3.7 Hz), 112.6 (q, *J* = 3.7 Hz), 105.6, 103.3, 52.6.

Methyl 2-(2,4-diaminophenoxy)-3-methoxy-5-propylbenzoate (**4e**)

Grayish oily (815 mg, 96%). HPLC analysis: 68.2%. m.p. None. ESI-MS *m*/*z*: 330.8 (M + H)^+^. ^1^H-NMR (400 MHz, CDCl_3_): 0.96 (t, *J* = 7.2 Hz, 3H), 1.64–1.72 (m, 2H), 2.60 (t, *J* = 7.6 Hz, 2H), 3.74 (s, 3H), 3.77 (s, 3H), 5.89 (dd, *J* = 8.4, 2.8 Hz, 1H), 6.17 (s, 1H), 6.18 (d, *J* = 12.0 Hz, 1H), 6.93 (d, *J* = 1.6 Hz, 1H), 7.24 (d, *J* = 2.0 Hz, 1H). ^13^C-NMR (100 MHz, CDCl_3_): 166.5, 153.0, 142.5, 141.3, 140.6, 139.6, 137.1, 125.6, 122.3, 116.7, 114.4, 104.9, 103.6, 56.4, 52.2, 37.8, 24.4, 13.8.

Methyl 2-(2,4-diaminophenoxy)-3,5-diisopropylbenzoate (**4f**)

Grayish-white solid (900 mg, 81.5%). HPLC analysis: 96.2%. m.p. 112–114 °C. ESI-MS *m*/*z*: 342.8 (M + H)^+^. ^1^H-NMR (400 MHz, CDCl_3_): 1.18 (d, *J* = 7.2 Hz, 6H), 1.27 (d, *J* = 6.8 Hz, 6H), 2.88–2.98 (m, 1H), 3.21–3.28 (m, 1H), 3.63 (br, s, 4H), 3.65 (s, 3H), 5.89 (dd, *J* = 8.4, 2.4 Hz, 1H), 6.03 (d, *J* = 8.4 Hz, 1H), 6.19 (d, *J* = 2.8 Hz, 1H), 7.33 (d, *J* = 2.4 Hz, 1H), 7.50 (d, *J* = 2.4 Hz, 1H). ^13^C-NMR (100 MHz, CDCl_3_): 166.9, 149.8, 145.1, 143.0, 141.2, 140.7, 136.8, 128.9, 126.7, 124.4, 113.7, 105.1, 103.7, 52.0, 33.8, 26.9, 24.0, 23.4.

Methyl 3,5-di-tert-butyl-2-(2,4-diaminophenoxy)benzoate (**4g**)

Gray solid (775 mg, 91%). HPLC analysis: 96.6%. m.p. 164–167 °C. ESI-MS *m*/*z*: 371.0 (M + H)^+^. ^1^H-NMR (400 MHz, CDCl_3_): 1.33 (s, 9H), 1.40 (s, 9H), 3.52 (s, 3H), 3.63 (s, 4H), 5.88 (dd, *J* = 8.4, 2.4 Hz, 1H), 6.10 (d, *J* = 8.8 Hz, 1H), 6.16 (d, *J* = 2.4 Hz, 1H), 7.49 (d, *J* = 2.4 Hz, 1H), 7.56 (d, *J* = 2.4 Hz, 1H). ^13^C-NMR (100 MHz, CDCl_3_): 168.0, 151.6, 146.0, 142.7, 141.3, 140.8, 137.6, 127.7, 125.8, 124.8, 114.9, 105.0, 103.3, 51.9, 35.4, 34.7, 31.4, 30.7.

#### 3.2.4. General Procedure for the Synthesis of Compounds **5a**–**5g**

Solution of **4a**–**4g** (120 mg, 0.46 mmol) in THF/H_2_O (5/1, 12 mL) was stirred, and 6 N hydrochloric acid (2 mL) was added under a nitrogen atmosphere. The reaction mixture was stirred at 70 °C under reflux overnight. After the reaction solution was cooled to room temperature, its pH was adjusted to neutral with 2 N sodium hydroxide (NaOH) solution. Then, the solvent was evaporated to dryness under reduced pressure. The resulting residue was dissolved in EA (20 mL), and the organic phase was washed with saturated brine (15 mL × 3) and dried over anhydrous Na_2_SO_4_. After removal of the solvent in a vacuum, the residue was purified by silica gel chromatography with PE/EA (1/1) to obtain compounds **5a**–**5g**.

8-aminodibenzo[b,f][1,4]oxazepin-11(10H)-one (**5a**)

Gray solid (70 mg, 67%). HPLC analysis: 95.3%. m.p. 248–251 °C. ESI-MS *m*/*z*: 226.8 (M + H)^+^. ^1^H-NMR (400 MHz, CD_3_OD): 6.47–6.50 (m, 2H), 7.02 (d, *J* = 12.4 Hz, 1H), 7.22–7.28 (m, 2H), 7.56 (td, *J* = 8.0, 2.0 Hz, 1H), 7.81 (dd, *J* = 7.6, 1.6 Hz, 1H). ^13^C-NMR (100 MHz, CD_3_OD): 168.1, 160.4, 145.5, 143.4, 134.1, 131.0, 130.8, 125.6, 124.6, 121.2, 120.2, 112.2, 107.2.

8-amino-4-methoxydibenzo[b,f][1,4]oxazepin-11(10H)-one (**5b**)

Gray solid (332.8 mg, 50%). HPLC analysis: 95.1%. m.p. 245–248 °C. ESI-MS *m*/*z*: 257.6 (M + H)^+^. ^1^H-NMR (400 MHz, *d*_6_-DMSO): 3.84 (s, 3H), 6.79 (dd, *J* = 8.4, 2.4 Hz, 1H), 6.86–6.89 (m, 3H), 7.24 (d, *J* = 8.8 Hz, 1H), 7.71 (d, *J* = 8.8 Hz, 1H), 10.4 (s, 1H). ^13^C-NMR (100 MHz, *d*_6_-DMSO): 166.0, 164.5, 160.8, 146.0, 137.1, 133.2, 132.5, 122.5, 118.2, 116.1, 112.2, 105.7, 56.3.

8-amino-4-methyldibenzo[b,f][1,4]oxazepin-11(10H)-one (**5c**)

Grayish-white solid (170 mg, 48%). HPLC analysis: 100.0%. m.p. 231–234 °C. ESI-MS *m*/*z*: 241.0 (M + H)^+^. ^1^H-NMR (400 MHz, *d*_6_-DMSO): 2.33 (s, 3H), 6.72 (dd, *J* = 8.4, 2.0 Hz, 1H), 6.79 (d, *J* = 2.0 Hz, 1H), 7.10 (d, *J* = 9.2 Hz, 1H), 7.11 (s, 1H), 7.17 (d, *J* = 8.4 Hz, 1H), 7.63 (d, *J* = 8.0 Hz, 1H), 10.5 (s, 1H). ^13^C-NMR (100 MHz, *d*_6_-DMSO): 166.4, 159.4, 145.7, 145.6, 138.3, 132.3, 131.7, 126.5, 123.3, 122.4, 121.1, 115.5, 111.5, 21.3.

8-amino-4-(trifluoromethyl)dibenzo[b,f][1,4]oxazepin-11(10H)-one (**5d**)

Gray solid (216.4 mg, 92%). HPLC analysis: 100.0%. m.p. 255–259 °C. ESI-MS *m*/*z*: 295.4 (M + H)^+^. ^1^H-NMR (400 MHz, d_6_-DMSO): 5.16 (s, 2H), 6.30–6.35 (m, 2H), 7.05 (d, *J* = 8.0 Hz, 1H), 7.62–7.67 (m, 2H), 7.95 (d, *J* = 7.2 Hz, 1H), 10.55 (s, 1H). ^13^C-NMR (100 MHz, *d*_6_-DMSO): 165.5, 160.1, 147.5, 141.2, 134.3, 133.9, 133.3, 131.1, 130.4, 122.0 (q, *J* = 3.5 Hz), 118.1 (q, *J* = 3.7 Hz), 110.9, 106.3.

8-amino-3-methoxy-5-propyldibenzo[b,f][1,4]oxazepin-11(10H)-one (**5e**)

White solid (140 mg, 25.8%). HPLC analysis: 100.0%. m.p. 119–122 °C. ESI-MS *m*/*z*: 298.7 (M + H)^+^. ^1^H-NMR (400 MHz, *d*_6_-DMSO): 0.87 (t, *J* = 7.2 Hz, 3H), 1.52–1.61 (m, 2H), 2.52 (t, *J* = 7.2 Hz, 2H), 3.89 (s, 3H), 5.07 (s, 2H), 6.25 (dd, *J* = 8.8, 2.8 Hz, 1H), 6.31 (d, *J* = 2.4 Hz, 1H), 6.87 (d, *J* = 8.4 Hz, 1H), 7.04 (d, *J* = 2.0 Hz, 1H), 7.08 (d, *J* = 3.2 Hz, 1H), 10.27 (s, 1H). ^13^C-NMR (100 MHz, *d*_6_-DMSO): 166.9, 150.6, 147.1, 146.9, 142.0, 139.5, 132.0, 127.3, 121.8, 121.4, 116.9, 110.4, 106.1, 56.7, 37.4, 24.5, 14.0.

8-amino-3,5-diisopropyldibenzo[b,f][1,4]oxazepin-11(10H)-one (**5f**)

White solid (510 mg, 80.3%). HPLC analysis: 100.0%. m.p. 224–227 °C. ESI-MS *m*/*z*: 311.0 (M + H)^+^. ^1^H-NMR (400 MHz, *d*_6_-DMSO): 1.17 (dd, *J* = 6.8 Hz, 6H), 1.24 (d, *J* = 6.8 Hz, 6H), 2.86–2.93 (m, 1H), 3.65–3.71 (s, 1H), 6.37 (dd, *J* = 8.4, 2.4 Hz, 1H), 6.41 (d, *J* = 2.8 Hz, 1H), 7.00 (d, *J* = 8.4 Hz, 1H), 7.38 (dd, *J* = 8.0, 2.0 Hz, 2H), 10.33 (s, 1H). ^13^C-NMR (100 MHz, *d*_6_-DMSO): 167.3, 154.9, 145.2, 144.9, 142.8, 139.8, 132.2, 129.1, 126.5, 126.3, 121.9, 111.6, 107.3, 33.4, 26.3, 24.3, 23.8.

8-amino-3,5-di-tert-butyldibenzo[b,f][1,4]oxazepin-11(10H)-one (**5g**)

White solid (152 mg, 37.8%). HPLC analysis: 100.0%. m.p. 228–232 °C. ESI-MS *m*/*z*: 339.0 (M + H)^+^. ^1^H-NMR (400 MHz, *d*_6_-DMSO): 1.26 (s, 9H), 1.50 (s, 9H), 5.11 (s, 2H), 6.26 (dd, *J* = 8.8, 2.8 Hz, 1H), 6.30 (d, *J* = 2.4 Hz, 1H), 7.07 (d, *J* = 8.8 Hz, 1H), 7.51 (d, *J* = 2.4 Hz, 1H), 7.55 (d, *J* = 2.4 Hz, 1H), 10.27 (s, 1H). ^13^C-NMR (100 MHz, *d*_6_-DMSO): 167.8, 157.6, 146.9, 146.0, 141.6, 140.2, 132.2, 128.5, 127.3, 126.8, 121.9, 110.1, 106.0, 35.4, 34.6, 31.7, 31.5.

#### 3.2.5. General Procedure for the Synthesis of Compounds **Ia**–**Ig**

5-chloroindole-2-carboxylic acid (1.2 eq) was dissolved in 6 mL of DMF. Under stirring, HATU (1.5 eq) and Et_3_N (4.6 eq) were sequentially added, and the mixture was stirred at room temperature for 10 min. Then **5a**–**5g** (1 eq) was added, and the mixture was heated to 45 °C and stirred overnight. After completion of the reaction, the mixture was cooled to room temperature and poured into ice water, where a large amount of solid precipitated. Vacuum filtration afforded a filter cake containing crude compounds **Ia**–**Ig**.

The filter cake was purified in two groups: the filter cake containing **Ia**–**Id** was recrystallized from acetone to obtain compounds **Ia**–**Id**; the filter cake containing **Ie**–**Ig** was first dissolved in a methanol-water mixed solvent (with an appropriate amount of DMSO added to assist dissolution) and then purified by C18 reverse-phase column chromatography with H_2_O/CH_3_OH (1/4) to obtain compounds **Ie**–**Ig**.

5-chloro-N-(11-oxo-10,11-dihydrodibenzo[b,f][1,4]oxazepin-8-yl)-1H-indole-2-carboxamide (**Ia**)

White solid (127 mg, 71.6%). HPLC analysis: 88.5%. m.p. 120–122 °C. ESI-MS *m*/*z*: 166.9 (M-H)^−^. ^1^H-NMR (400 MHz, CDCl_3_): 3.92 (s, 3H), 5.79 (s, 1H), 6.37–6.41 (m, 1H), 7.73 (d, *J* = 8.8 Hz, 1H), 11.01 (s, 1H). ^13^C-NMR (100 MHz, CDCl_3_): 170.4, 163.5, 162.0, 132.0, 107.9, 105.9, 103.1, 52.1.

5-chloro-N-(4-methoxy-11-oxo-10,11-dihydrodibenzo[b,f][1,4]oxazepin-8-yl)-1H-indole-2-carboxamide (**Ib**)

Pale yellow solid (85 mg, 59%). HPLC analysis: 82.8%. m.p. 317–320 °C. ESI-MS *m*/*z*: 432.3 (M-H)^−^. ^1^H-NMR (400 MHz, *d*_6_-DMSO): 3.85 (s, 3H), 6.88–6.91 (m, 2H), 7.23 (dd, *J* = 8.7, 1.8 Hz, 1H), 7.32 (d, *J* = 8.7 Hz, 1H), 7.42 (s, 1H), 7.49 (dd, *J* = 12.0, 5.5 Hz, 2H), 7.73 (d, *J* = 8.6 Hz, 2H), 7.78 (s, 1H), 10.39 (s, 1H), 10.48 (s, 1H), 11.96 (s, 1H). ^13^C-NMR (100 MHz, *d*_6_-DMSO): 165.6, 164.0, 160.3, 159.3, 145.9, 136.4, 135.2, 132.7, 131.3, 128.0, 124.4, 123.9, 121.3, 120.8, 117.7, 116.7, 114.0, 113.1, 111.7, 105.2, 103.5, 55.8.

5-chloro-N-(4-methyl-11-oxo-10,11-dihydrodibenzo[b,f][1,4]oxazepin-8-yl)-1H-indole-2-carboxamide (**Ic**)

Pale yellow solid (69 mg, 50%). HPLC analysis: 84.1%. m.p. 346–348 °C. ESI-MS *m*/*z*: 416.4 (M-H)^−^. ^1^H-NMR (400 MHz, *d*_6_-DMSO): 2.37 (s, 3H), 7.14 (d, *J* = 8.0 Hz, 1H), 7.17 (s, 1H), 7.23 (dd, *J* = 7.6, 2.0 Hz, 1H), 7.31 (d, *J* = 8.8 Hz, 1H), 7.42 (d, *J* = 1.2 Hz, 1H), 7.47–7.51 (m, 2H), 7.68 (d, *J* = 8.0 Hz, 1H), 7.73 (d, *J* = 2.4 Hz, 1H), 7.78 (d, *J* = 2.0 Hz, 1H), 10.40 (s, 1H), 10.59 (s, 1H), 11.98 (s, 1H). ^13^C-NMR (100 MHz, *d*_6_-DMSO): 166.4, 159.8, 159.3, 146.7, 145.6, 136.8, 135.7, 133.2, 131.74, 131.70, 128.5, 126.5, 124.9, 124.4, 123.3, 121.8, 121.3, 121.2, 117.3, 114.5, 113.6, 104.0, 21.3.

5-chloro-N-(11-oxo-4-(trifluoromethyl)-10,11-dihydrodibenzo[b,f][1,4]oxazepin-8-yl)-1H-indole-2-carboxamide (**Id**)

White solid (80 mg, 53%). HPLC analysis: 100.0%. m.p. 322–324 °C. ESI-MS *m*/*z*: 470.4 (M-H)^−^. ^1^H-NMR (300 MHz, *d*_6_-DMSO): 7.23 (d, *J* = 8.1 Hz, 1H), 7.42–7.55 (m, 4H), 7.70 (d, *J* = 7.7 Hz, 1H), 7.79 (s, 3H), 8.02 (d, *J* = 7.5 Hz, 1H), 10.39 (s, 1H), 10.88 (s, 1H), 11.93 (s, 1H). ^13^C-NMR (75 MHz, *d*_6_-DMSO): 167.8, 162.4, 162.1, 149.0, 139.9, 138.3, 136.9 (d, *J* = 33.0 Hz), 136.0, 135.7, 133.7, 132.7, 131.1, 127.5, 127.0, 125.1 (d, *J* = 3.15 Hz), 124.6, 123.9, 121.0 (d, *J* = 2.78 Hz), 120.2, 117.0, 116.4, 106.7.

5-chloro-N-(3-methoxy-11-oxo-5-propyl-10,11-dihydrodibenzo[b,f][1,4]oxazepin-8-yl)-1H-indole-2-carboxamide (**Ie**)

White solid (65 mg, 49%). HPLC analysis: 84.1%. m.p. 287–289 °C. ESI-MS *m*/*z*: 474.2 (M-H)^−^. ^1^H-NMR (400 MHz, *d*_6_-DMSO): 0.88 (s, 3H), 1.59 (s, 2H), 3.38 (s, 2H), 3.90 (s, 3H), 7.13 (d, *J* = 23.1 Hz, 2H), 7.24 (s, 2H), 7.42 (s, 1H), 7.49 (s, 2H), 7.76 (d, *J* = 13.9 Hz, 2H), 10.45 (s, 1H), 10.65 (s, 1H), 12.03 (s, 1H). ^13^C-NMR (100 MHz, *d*_6_-DMSO): 166.6, 159.7, 150.7, 147.1, 146.3, 140.1, 136.7, 135.7, 133.2, 132.0, 128.5, 127.0, 124.9, 124.4, 121.8, 121.4, 121.3, 117.2, 114.5, 113.6, 104.1, 56.8, 37.4, 24.5, 14.1.

5-chloro-N-(3,5-diisopropyl-11-oxo-10,11-dihydrodibenzo[b,f][1,4]oxazepin-8-yl)-1H-indole-2-carboxamide (**If**)

White solid (85 mg, 67%). HPLC analysis: 97.6%. m.p. 299–302 °C. ESI-MS *m*/*z*: 486.1 (M-H)^−^. ^1^H-NMR (400 MHz, *d*_6_-DMSO): 1.23 (d, *J* = 35.2 Hz, 12H), 2.89–2.97 (m, 1H), 3.70- 3.81 (m, 1H), 7.29 (d, *J* = 43.3 Hz, 2H), 7.34–7.57 (m, 5H), 7.75 (d, *J* = 18.6 Hz, 2H), 10.40 (s, 1H), 10.61 (s, 1H), 11.97 (s, 1H). ^13^C-NMR (100 MHz, *d*_6_-DMSO): 167.1, 159.7, 154.5, 147.1, 145.6, 140.0, 136.7, 135.7, 133.2, 132.1, 129.3, 128.5, 126.4, 126.3, 124.9, 124.4, 121.8, 121.3, 117.3, 114.5, 113.6, 104.1, 33.4, 26.3, 24.3, 23.8.

5-chloro-N-(3,5-di-tert-butyl-11-oxo-10,11-dihydrodibenzo[b,f][1,4]oxazepin-8-yl)-1H-indole-2-carboxamide (**Ig**)

White solid (85 mg, 72%). HPLC analysis: 100.0%. m.p. 326–329 °C. ESI-MS *m*/*z*: 514.1 (M-H)^−^. ^1^H-NMR (400 MHz, *d*_6_-DMSO): 1.29 (s, 9H), 1.56 (s, 9H), 7.24 (s, 1H), 7.48 (d, *J* = 15.6 Hz, 4H), 7.68 (dd, *J* = 63.2, 14.1 Hz, 4H), 10.44 (s, 1H), 10.62 (s, 1H), 11.99 (s, 1H). ^13^C-NMR (100 MHz, *d*_6_-DMSO): 167.5, 159.8, 156.9, 146.9, 146.6, 140.5, 136.8, 135.7, 133.2, 132.1, 129.0, 128.5, 127.0, 124.9, 124.4, 121.9, 121.3, 116.8, 114.4, 113.5, 104.1, 35.5, 34.7, 31.7, 31.5.

#### 3.2.6. General Procedure for Synthesis of Compounds **IIa**–**IIf**

Compounds **IIa**–**IIf** were synthesized using the same procedure as for **Ia**, replacing 5-chloroindole-2-carboxylic acid with the following aryl acids: 5-fluoroindole-2-carboxylic acid (a), 5-hydroxyindole-2-carboxylic acid (b), 5-methoxyindole-2-carboxylic acid (c), 5-methoxy-1H-pyrrolo[2,3-b]pyridine-2-carboxylic acid (d), 4-methylindole-2-carboxylic acid (e), and 5-methylindole-2-carboxylic acid (f).

N-(11-oxo-4-(trifluoromethyl)-10,11-dihydrodibenzo[b,f][1,4]oxazepin-8-yl)-5-fluoro-1H-indole-2-carboxamide (**IIa**)

Pale yellow solid (42 mg, 36.9%). HPLC analysis: 100.0%. m.p. 324–326 °C. ESI-MS *m*/*z*: None. ^1^H-NMR (300 MHz, *d*_6_-DMSO): 7.10 (td, *J* = 9.3, 2.4 Hz, 1H), 7.42–7.49 (m, 4H), 7.54 (dd, *J* = 8.8, 2.3 Hz, 1H), 7.70 (d, *J* = 7.8 Hz, 1H), 7.80 (s, 2H), 8.01 (d, *J* = 8.0 Hz, 1H), 10.40 (s, 1H), 10.90 (s, 1H), 11.87 (s, 1H). ^13^C-NMR (75 MHz, *d*_6_-DMSO): 167.8, 162.4, 162.1, 160.3 (d, *J* = 231.8 Hz), 149.0, 139.9, 136.6, 136.0, 135.9, 133.7, 132.7, 125.1, 124.5, 121.0, 120.2, 116.7, 116.6, 116.4, 115.9, 115.5, 109.1, 108.8, 107.2 (d, *J* = 4.8 Hz).

5-hydroxy-N-(11-oxo-4-(trifluoromethyl)-10,11-dihydrodibenzo[b,f][1,4]oxazepin-8-yl)-1H-indole-2-carboxamide (**IIb**)

Brownish-red solid (30 mg, 26.5%). HPLC analysis: 100.0%. m.p. None. ESI-MS *m*/*z*: None. ^1^H-NMR (300 MHz, *d*_6_-DMSO): 6.79 (dd, *J* = 8.7, 2.1 Hz, 1H), 6.93 (d, *J* = 1.7 Hz, 1H), 7.22 (s, 1H), 7.28 (d, *J* = 8.8 Hz, 1H), 7.42 (d, *J* = 8.8 Hz, 1H), 7.52 (dd, *J* = 8.9, 2.2 Hz, 1H), 7.70 (d, *J* = 8.2 Hz, 1H), 7.79 (s, 2H), 8.01 (d, *J* = 8.1 Hz, 1H), 8.84 (s, 1H), 10.22 (s, 1H), 10.89 (s, 1H), 11.44 (s, 1H). ^13^C-NMR (75 MHz, *d*_6_-DMSO): 167.8, 162.8, 162.1, 154.3, 148.8, 140.1, 136.0, 134.8, 134.3, 133.7, 132.7, 130.7, 125.1, 124.5, 121.0, 120.1, 118.3, 116.3, 115.9, 107.5, 106.3.

5-methoxy-N-(11-oxo-4-(trifluoromethyl)-10,11-dihydrodibenzo[b,f][1,4]oxazepin-8-yl)-1H-indole-2-carboxamide (**IIc**)

White solid (59 mg, 52.7%). HPLC analysis: 100.0%. m.p. 294–297 °C. ESI-MS *m*/*z*: None. ^1^H-NMR (500 MHz, *d*_6_-DMSO): 3.79 (s, 3H), 6.89 (dd, *J* = 8.8, 1.8 Hz, 1H), 7.14 (s, 1H), 7.33 (s, 1H), 7.37 (d, *J* = 8.9 Hz, 1H), 7.42 (d, *J* = 8.7 Hz, 1H), 7.53 (dd, *J* = 8.6, 1.7 Hz, 1H), 7.70 (d, *J* = 8.2 Hz, 1H), 7.79 (s, 2H), 8.01 (d, *J* = 8.0 Hz, 1H), 10.26 (s, 1H), 10.87 (s, 1H), 11.57 (s, 1H). ^13^C-NMR (125 MHz, *d*_6_-DMSO): 165.2, 160.1, 159.5, 154.4, 146.3, 137.5, 134.5, 134.2, 133.4, 132.7, 131.9, 131.1, 130.1, 127.8, 122.5 (q, *J* = 2.9 Hz), 121.9, 118.4 (q, *J* = 3.3 Hz), 117.6, 115.6, 113.8, 113.7, 104.3, 102.7, 55.8.

5-methoxy-N-(11-oxo-4-(trifluoromethyl)-10,11-dihydrodibenzo[b,f][1,4]oxazepin-8-yl)-1H-pyrrolo [2,3-b]pyridine-2-carboxamide (**IId**)

White solid (55 mg, 47%). HPLC analysis: 100.0%. m.p. None. ESI-MS *m*/*z*: None. ^1^H-NMR (300 MHz, *d*_6_-DMSO): 11.93 (s, 1H), 10.91 (s, 1H), 10.51 (s, 1H), 8.48 (s, 1H), 8.01 (d, *J* = 8.1 Hz, 1H), 7.80 (s, 2H), 7.71 (d, *J* = 8.0 Hz, 1H), 7.54 (dd, *J* = 8.8, 2.1 Hz, 1H), 7.44 (d, *J* = 8.8 Hz, 1H), 7.30 (s, 1H), 7.01 (s, 1H), 3.86 (s, 3H). ^13^C-NMR (75 MHz, *d*_6_-DMSO): 167.7, 162.2, 162.0, 160.6, 149.2, 139.6, 139.2, 138.4, 137.2, 136.7, 136.0, 135.0, 134.0, 132.7, 125.1, 124.6, 121.0, 120.4, 116.5, 104.8, 100.7, 56.7.

4-methyl-N-(11-oxo-4-(trifluoromethyl)-10,11-dihydrodibenzo[b,f][1,4]oxazepin-8-yl)-1H-indole-2-carboxamide (**IIe**)

White solid (50 mg, 44.6%). HPLC analysis: 100.0%. m.p. 357–359 °C. ESI-MS *m*/*z*: None. ^1^H-NMR (500 MHz, *d*_6_-DMSO): 2.54 (s, 3H), 6.87 (d, *J* = 6.9 Hz, 1H), 7.12 (t, *J* = 7.6 Hz, 1H), 7.30 (d, *J* = 8.2 Hz, 1H), 7.43 (d, *J* = 8.8 Hz, 1H), 7.48 (s, 1H), 7.55 (d, *J* = 8.8 Hz, 1H), 7.70 (d, *J* = 8.0 Hz, 1H), 7.79 (s, 1H), 7.83 (s, 1H), 8.01 (d, *J* = 8.0 Hz, 1H), 10.34 (s, 1H), 10.89 (s, 1H), 11.71 (s, 1H). ^13^C-NMR (125 MHz, *d*_6_-DMSO): 165.2, 160.2, 159.5, 146.3, 137.6, 137.2, 134.5, 134.2, 133.4, 131.2, 131.1, 131.0, 130.2, 127.7, 124.5, 122.5 (q, *J* = 2.1 Hz), 121.9, 120.3, 118.4 (q, *J* = 3.3 Hz), 117.5, 113.7, 110.5, 103.4, 19.0.

5-methyl-N-(11-oxo-4-(trifluoromethyl)-10,11-dihydrodibenzo[b,f][1,4]oxazepin-8-yl)-1H-indole-2-carboxamide (**IIf**)

Pale yellow solid (40 mg, 44.4%). HPLC analysis: 100.0%. m.p. 318–321 °C. ESI-MS *m*/*z*: None. ^1^H-NMR (500 MHz, *d*_6_-DMSO): 2.39 (s, 3H), 7.06 (d, *J* = 8.2 Hz, 1H), 7.34 (s, 1H), 7.37 (d, *J* = 8.4 Hz, 1H), 7.42 (d, *J* = 8.8 Hz, 1H), 7.45 (s, 1H), 7.53 (dd, *J* = 8.8, 2.3 Hz, 1H), 7.70 (d, *J* = 8.2 Hz, 1H), 7.80 (d, *J* = 6.8 Hz, 2H), 8.01 (d, *J* = 8.1 Hz, 1H), 10.26 (s, 1H), 10.87 (s, 1H), 11.58 (s, 1H). ^13^C-NMR (125 MHz, *d*_6_-DMSO): 165.2, 160.2, 159.5, 146.3, 137.5, 135.8, 134.5, 134.2, 133.4, 131.6, 131.1, 130.1, 129.0, 127.7, 126.2, 122.5 (q, *J* = 3.6 Hz), 121.9, 121.4, 118.4 (q, *J* = 3.8 Hz), 117.5, 113.7, 112.6, 104.1, 21.6.

### 3.3. In Vitro Experiments

#### Enzyme Activity Assay

The inhibitory activity of the test compounds against RMGPa was evaluated using a microplate reader (Bio-Rad Laboratories, Berkeley, CA, USA), based on the release of inorganic phosphate during glycogenolysis [26]. First, the test compounds were dissolved in DMSO and diluted to various concentrations for IC_50_ determination. A 100 μL aliquot of buffer, containing 50 mM Hepes (pH = 7.2), 100 mM potassium chloride (KCl), 2.5 mM magnesium chloride (MgCl_2_), 0.5 mM glucose-1-phosphate, 1 mg/mL glycogen, and the test compound solution, was added to a 96-well Costar microplate (Corning Inc., New York, NY, USA). The reaction mixture was incubated at 22 °C for 25 min, after which 150 μL of solution containing 10 mg/mL ammonium molybdate and 0.38 mg/mL malachite green was added to stop the reaction. The absorbance of phosphate was measured at 620 nm. IC_50_ values were determined by fitting the inhibition data to a dose–response curve using a logistic derivative equation.

In the activity assays, all compounds exhibited typical dose-dependent inhibition curves, and reasonable structure–activity relationships were observed across the series, which largely reduces the possibility of artifactual inhibition due to non-specific compound aggregation.

### 3.4. In Vivo Experiments

#### 3.4.1. Animals

C57BL/6J mice were used in this study. All animals were housed under standard conditions (23–25 °C, 30–70% relative humidity, 12 h light/dark cycle) with free access to food and sterilized water. The standard chow was procured from Beijing HFK Bioscience Co., Ltd. (Beijing, China), and the high-fat diet (60% Kcal fat, D12492) was from Research Diets, Inc. (New Brunswick, NJ, USA). All animal procedures were approved by the Institutional Animal Care and Use Committee (IACUC) of Chengde Medical University and were conducted in an AAALAC International-accredited facility in accordance with its guidelines. For details, please see the ‘Institutional Review Board Statement’ section.

#### 3.4.2. Materials

Metformin hydrochloride, Shanghai Demer Pharmaceutical Technology Co., Ltd. (Shanghai, China); Sodium carboxymethyl cellulose (CMC-Na), Fuchen Chemical Reagent Co., Ltd. (Tianjin, China); Dimethyl sulfoxide (DMSO), Polyethylene glycol 400 (PEG400), and Chloral hydrate, China National Medicines Co., Ltd. (Beijing, China); Epinephrine Hydrochloride Injection (1 mg:1 mL), Tianjin Jinyao Pharmaceutical Co., Ltd. (Tianjin, China); Blood glucose meter (JPS-5), Beijing Yicheng Bioelectronic Technology Co., Ltd. (Beijing, China); Analytical balance (Model Discovery DV215CD), OHAUS Corp. (Shanghai, China); Manual Adjustable pipettes (Dragon TopPette series), DLAB Scientific Co., Ltd. (Beijing, China); Ultrasonic cleaner, Ningbo Scientz Biotechnology Co., Ltd. (Ningbo, China).

#### 3.4.3. Acute and Chronic Hypoglycemic Experiments

The animal experiments were conducted following the method described in reference [23]. Male C57BL/6J mice (6–8 weeks old, 18–20 g) were obtained from Beijing Huafukang Biotechnology Co., Ltd. (Beijing, China), maintained under standard conditions (food/water ad libitum), and acclimatized for 1 week. After acclimatization, baseline blood glucose was measured via tail vein sampling. Subsequently, mice were randomized into 6 groups (10/group) by blood glucose/body weight, with no significant differences in initial blood glucose (mean ± SEM). Post-grouping, daily oral 0.5% CMC-Na (as vehicle) was given. On the night before the experimental day, mice were fasted overnight with free access to water. On the experimental day, mice were orally administered Compound Id (50, 25, 12.5 mg/kg), metformin (400 mg/kg, as positive control), or 0.5% CMC-Na, followed by subcutaneous 0.2 mg/kg adrenaline to induce hyperglycemia. Finally, blood glucose was measured/recorded at 0, 30, 60, 90, and 120 min post-administration via JPS-5 meter.

The animal experiments of the DIO mouse model were also performed according to the previous literature [27]. Male C57BL/6J mice (3 weeks old, 7–9 g) were purchased from Beijing Huafukang Biotechnology Co., Ltd. (Beijing, China), and housed under standard conditions (food/water ad libitum). After 1 week of acclimatization, baseline blood glucose was measured via tail vein sampling. Mice were then randomized into 6 groups (10/group) by blood glucose/body weight (no significant differences in initial blood glucose, mean ± SEM): normal controls on normal chow, others fed a high-fat diet for 15 weeks to establish a DIO model. On the night before the experiment, mice were fasted overnight with free access to water. On the experimental day, mice were orally given Compound Id (50, 25, 12.5 mg/kg), metformin (400 mg/kg), or 0.5% CMC-Na (10 mL/kg) once daily for 4 weeks. Tail vein blood was collected on days 0, 7, 14, 21, and 28; 6 h fasting blood glucose was measured via JPS-5 meter.

### 3.5. Statistical Analysis Statistical

Statistical analysis was performed using SPSS Statistics 17.0. Data are presented as mean ± SD (in vitro) or mean ± SEM (in vivo). Student’s *t*-test was used for two-group comparisons, one-way ANOVA for multiple groups at single time points, and repeated measures ANOVA for longitudinal DIO data. All data from animals that completed the study were included, as no outliers were identified. Sample sizes were based on established conventions for the models used, and *p* < 0.05 was considered significant.

## 4. Conclusions

In this study, a series of dibenzoxazepinone derivatives was designed and synthesized, and their GP inhibitory activities and hypoglycemic activities in hyperglycemic mice were screened. The results showed that most of the designed compounds had significant GP inhibitory activity, among which compound **Id** had the best activity, superior to the positive control drug **PSN-357**. More importantly, compound **Id** could significantly reduce the blood glucose levels of adrenaline-induced hyperglycemic mice and DIO mice. In conclusion, dibenzoxazepinone derivatives are a new type of GP inhibitors and thus have the potential to be developed as novel hypoglycemic drugs.

## Figures and Tables

**Table 1:** Inhibition test of RMGPa for novel dibenzooxazepinone derivatives.

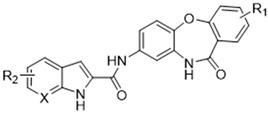				
Compound	X	R_1_	R_2_	IC_50_ ^a^ (nM)
**Ia**	C	H	5-Cl	NI ^b^
**Ib**	C	4-OCH_3_	5-Cl	291 ± 89
**Ic**	C	4-CH_3_	5-Cl	NI
**Id**	C	4-CF_3_	5-Cl	266 ± 1
**Ie**	C	3-OCH_3_,5-CH_2_CHCH_2_	5-Cl	2178 ± 156
**If**	C	3-CH(CH_3_)_2_,5-CH(CH_3_)_2_	5-Cl	NI
**Ig**	C	3-C(CH_3_)_3_,5-C(CH_3_)_3_	5-Cl	NI
**IIa**	C	4-CF_3_	5-F	942 ± 60
**IIb**	C	4-CF_3_	5-OH	723 ± 82
**IIc**	C	4-CF_3_	5-OCH_3_	840 ± 50
**IId**	N	4-CF_3_	5-OCH_3_	1790 ± 104
**IIe**	C	4-CF_3_	4-CH_3_	NI
**IIf**	C	4-CF_3_	5-CH_3_	338 ± 55
**1 ^c^**	-	-	-	250 ± 50
**PSN-357**	-	-	-	420 ± 10

^a^ Each value represents the mean ± S.D. of three determinations. ^b^ NI means no inhibition. ^c^ Compound **1** is the parent compound [23].

## Data Availability

The data presented in this study are available on request from the corresponding author.

## Supplementary Materials

The following supporting information can be downloaded at: https://www.mdpi.com/article/10.3390/molecules30244797/s1.

## References

[1] Sun H., Saeedi P., Karuranga S., Pinkepank M., Ogurtsova K., Duncan B.B., Stein C., Basit A., Chan J.C.N., Mbanya J.C. (2022). IDF Diabetes Atlas: Global, regional and country-level diabetes prevalence estimates for 2021 and projections for 2045. Diabetes Res. Clin. Pract..

[2] Ahmad E., Lim S., Lamptey R., Webb D.R., Davies M.J. (2022). Type 2 diabetes. Lancet.

[3] Zhang B., Johnson M.M., Yuan T., Nguyen T.N., Okada J., Yang F., Xiaoli A.M., Melikian L.H., Xu S., Dadpey B. (2025). Hepatic glycogen directly regulates gluconeogenesis through an AMPK/CRTC2 axis in mice. J. Clin. Investig..

[4] Ma R., Du B., Shi C., Wang L., Zeng F., Han J., Guan H., Wang Y., Yan K. (2025). Molecular basis for the regulation of human phosphorylase kinase by phosphorylation and Ca^2+^. Nat. Commun..

[5] Agius L. (2015). Role of glycogen phosphorylase in liver glycogen metabolism. Mol. Aspects Med..

[6] Klabunde T., Wendt K.U., Kadereit D., Brachvogel V., Burger H.J., Herling A.W., Oikonomakos N.G., Kosmopoulou M.N., Schmoll D., Sarubbi E. (2005). Acyl ureas as human liver glycogen phosphorylase inhibitors for the treatment of type 2 diabetes. J. Med. Chem..

[7] Rines A.K., Sharabi K., Tavares C.D., Puigserver P. (2016). Targeting hepatic glucose metabolism in the treatment of type 2 diabetes. Nat. Rev. Drug Discov..

[8] Goyard D., Kónya B., Czifrák K., Larini P., Demontrond F., Leroy J., Balzarin S., Tournier M., Tousch D., Petit P. (2020). Glucose-based spiro-oxathiazoles as in vivo anti-hyperglycemic agents through glycogen phosphorylase inhibition. Org. Biomol. Chem..

[9] Drakou C.E., Gardeli C., Tsialtas I., Alexopoulos S., Mallouchos A., Koulas S.M., Tsagkarakou A.S., Asimakopoulos D., Leonidas D.D., Psarra A.G. (2020). Affinity Crystallography Reveals Binding of Pomegranate Juice Anthocyanins at the Inhibitor Site of Glycogen Phosphorylase: The Contribution of a Sugar Moiety to Potency and Its Implications to the Binding Mode. J. Agric. Food Chem..

[10] Alexopoulos S., McGawley M., Mathews R., Papakostopoulou S., Koulas S., Leonidas D.D., Zwain T., Hayes J.M., Skamnaki V. (2024). Evidence for the Quercetin Binding Site of Glycogen Phosphorylase as a Target for Liver-Isoform-Selective Inhibitors against Glioblastoma: Investigation of Flavanols Epigallocatechin Gallate and Epigallocatechin. J. Agric. Food Chem..

[11] Rocha S., Aniceto N., Guedes R.C., Albuquerque H.M.T., Silva V.L.M., Silva A.M.S., Corvo M.L., Fernandes E., Freitas M. (2022). An In Silico and an In Vitro Inhibition Analysis of Glycogen Phosphorylase by Flavonoids, Styrylchromones, and Pyrazoles. Nutrients.

[12] Tsitsanou K.E., Hayes J.M., Keramioti M., Mamais M., Oikonomakos N.G., Kato A., Leonidas D.D., Zographos S.E. (2013). Sourcing the affinity of flavonoids for the glycogen phosphorylase inhibitor site via crystallography, kinetics and QM/MM-PBSA binding studies: Comparison of chrysin and flavopiridol. Food Chem. Toxicol..

[13] Homolya L., Mathomes R.T., Varga L., Docsa T., Juhász L., Hayes J.M., Somsák L. (2024). Synthesis, In Silico and Kinetics Evaluation of N-(β-d-glucopyranosyl)-2-arylimidazole-4(5)-carboxamides and N-(β-d-glucopyranosyl)-4(5)-arylimidazole-2-carboxamides as Glycogen Phosphorylase Inhibitors. Int. J. Mol. Sci..

[14] Kun S., Begum J., Kyriakis E., Stamati E.C.V., Barkas T.A., Szennyes E., Bokor É., Szabó K.E., Stravodimos G.A., Sipos Á. (2018). A multidisciplinary study of 3-(β-d-glucopyranosyl)-5-substituted-1,2,4-triazole derivatives as glycogen phosphorylase inhibitors: Computation, synthesis, crystallography and kinetics reveal new potent inhibitors. Eur. J. Med. Chem..

[15] Mavreas K.F., Neofytos D.D., Chrysina E.D., Venturini A., Gimisis T. (2020). Synthesis, Kinetic and Conformational Studies of 2-Substituted-5-(β-d-glucopyranosyl)-pyrimidin-4-ones as Potential Inhibitors of Glycogen Phosphorylase. Molecules.

[16] Kun S., Mathomes R.T., Docsa T., Somsák L., Hayes J.M. (2023). Design and Synthesis of 3-(β-d-Glucopyranosyl)-4-amino/4-guanidino Pyrazole Derivatives and Analysis of Their Glycogen Phosphorylase Inhibitory Potential. Molecules.

[17] Galal S.A., Khattab M., Andreadaki F., Chrysina E.D., Praly J.P., Ragab F.A.F., El Diwani H.I. (2016). Synthesis of (benzimidazol-2-yl)aniline derivatives as glycogen phosphorylase inhibitors. Bioorg. Med. Chem..

[18] Szennyes E., Bokor É., Docsa T., Sipos Á., Somsák L. (2019). Synthesis of C-β-d-glucopyranosyl derivatives of some fused azoles for the inhibition of glycogen phosphorylase. Carbohydr. Res..

[19] Zhou J., Bie J., Wang X., Liu Q., Li R., Chen H., Hu J., Cao H., Ji W., Li Y. (2020). Discovery of N-Arylsulfonyl-Indole-2-Carboxamide Derivatives as Potent, Selective, and Orally Bioavailable Fructose-1,6-Bisphosphatase Inhibitors-Design, Synthesis, In Vivo Glucose Lowering Effects, and X-ray Crystal Complex Analysis. J. Med. Chem..

[20] Martin W.H., Hoover D.J., Armento S.J., Stock I.A., McPherson R.K., Danley D.E., Stevenson R.W., Barrett E.J., Treadway J.L. (1998). Discovery of a human liver glycogen phosphorylase inhibitor that lowers blood glucose in vivo. Proc. Natl. Acad. Sci. USA.

[21] Onda K., Shiraki R., Ogiyama T., Yokoyama K., Momose K., Katayama N., Orita M., Yamaguchi T., Furutani M., Hamada N. (2008). Design, synthesis, and pharmacological evaluation of N-bicyclo-5-chloro-1H-indole-2-carboxamide derivatives as potent glycogen phosphorylase inhibitors. Bioorg. Med. Chem..

[22] Oikonomakos N.G., Skamnaki V.T., Tsitsanou K.E., Gavalas N.G., Johnson L.N. (2000). A new allosteric site in glycogen phosphorylase b as a target for drug interactions. Structure.

[23] Wang Y., Yan Z., Guo Y., Zhang L. (2021). Discovery and evaluation of novel benzazepinone derivatives as glycogen phosphorylase inhibitors with potent activity. Future Med. Chem..

[24] Dachineni R., Kumar D.R., Callegari E., Kesharwani S.S., Sankaranarayanan R., Seefeldt T., Tummala H., Bhat G.J. (2017). Salicylic acid metabolites and derivatives inhibit CDK activity: Novel insights into aspirin’s chemopreventive effects against colorectal cancer. Int. J. Oncol..

[25] Hong S.J., Lee J.H., Kim E.J., Yang H.J., Park J.S., Hong S.K. (2017). Anti-Obesity and Anti-Diabetic Effect of Neoagarooligosaccharides on High-Fat Diet-Induced Obesity in Mice. Mar. Drugs.

[26] Webb M.R. (1992). A continuous spectrophotometric assay for inorganic phosphate and for measuring phosphate release kinetics in biological systems. Proc. Natl. Acad. Sci. USA.

[27] Wang Y., Li S., Yan Z., Guo Y., Yang D., Zhang L. (2022). Long-term pharmacokinetic and pharmacological evaluations of a novel indole-benzazepinone derivative on obese Type 2 diabetes mellitus. Future Med. Chem..

