# Supplementary material for: Discovery and Activity Evaluation of Novel Dibenzoxazepinone Derivatives as Glycogen Phosphorylase Inhibitors
Source: Molecules. 2025 Dec 16;30(24):4797. doi: 10.3390/molecules30244797 (PMC12735688; doi:10.3390/molecules30244797)
Supplement: Supplementary file 1 [file molecules-30-04797-s001.zip › molecules-4014361-supplementary.pdf]

Supplementary Information(SI)

A: Supplementary table

Table **S1.** HPLC purity data for representative compounds.

B:Supplementary figures

Figures **B1** to **B32**. HPLC purity data for representative compounds.

A: Supplementary table

Table S1. HPLC purity data for representative compounds (detected at 254 nm, DAD1 A).

Compound	Peak Area (%)	Retention Time (t, min)	Compound	Peak Area (%)	Retention Time (t, min)
**2a**	100%	13.6	**5d**	100%	13.4
**3a**	100%	16	**Id**	100%	17.6
**4a**	97.5%	18.6	**3e**	100%	18.1
**5a**	95.3%	17	**4e**	68.2%	20.3
**Ia**	88.5%	18.2	**5e**	100%	11.7
**3b**	100%	16.8	**3f**	96.4%	20.3
**4b**	55.4%	19	**4f**	96.2%	21.4
**5b**	95.1%	18.6	**5f**	100%	18.8
**Ib**	82.8%	18.5	**If**	97.6%	21
**3c**	97.4%	17.3	**3g**	97.1%	21
**4c**	87.1%	19.4	**5g**	100%	22
**5c**	100%	8.1	**Ig**	100%	21.8
**Ic**	84.1%	18.9	**IIa**	100%	19
**2d**	100%	18.1	**IIc**	100%	12.9
**3d**	100%	18	**IIe**	100%	17.5
**4d**	92.7%	20.1	**IIf**	100%	19.4

B:Supplementary figures

Figures B1 to **B32**. HPLC purity data for representative compounds.

Figure B1. Purity of compound **2a**

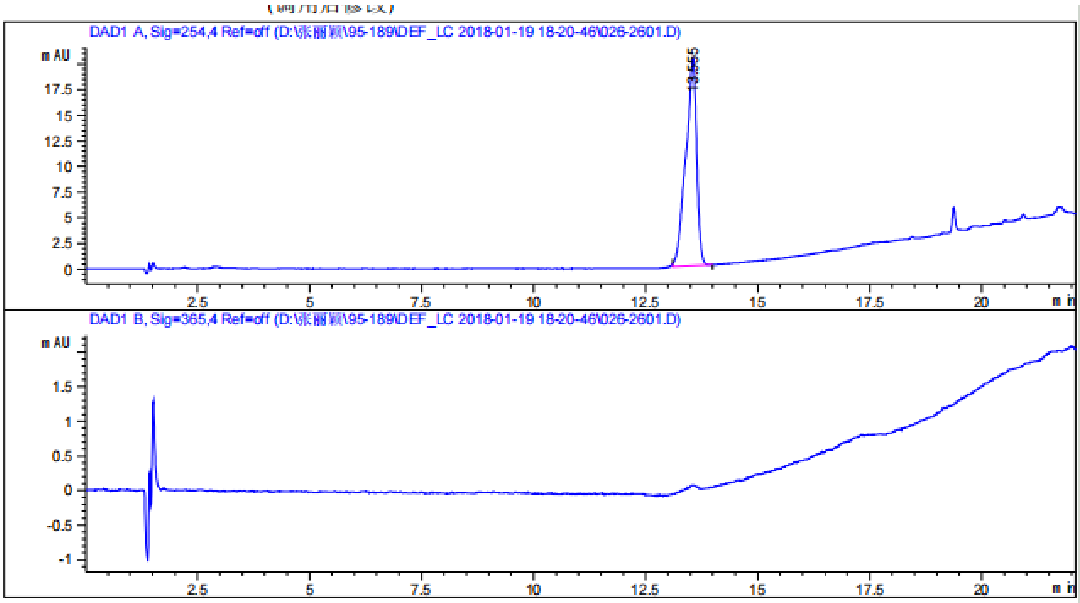

===

面积百分比报告

===

排序 : 信号
乘积因子 : 1.0000
稀释因子 : 1.0000
内标使用乘积因子和稀释因子

信号 1: DAD1 A, Sig=254,4 Ref=off

峰 #	保留时间 [min]	类型	峰宽 [min]	峰面积 [mAU*s]	峰高 [mAU]	峰面积 %
1	13.555	BB	0.2342	340.32004	20.23837	100.0000

总量 : 340.32004 20.23837

Figure B2. Purity of compound **3a**

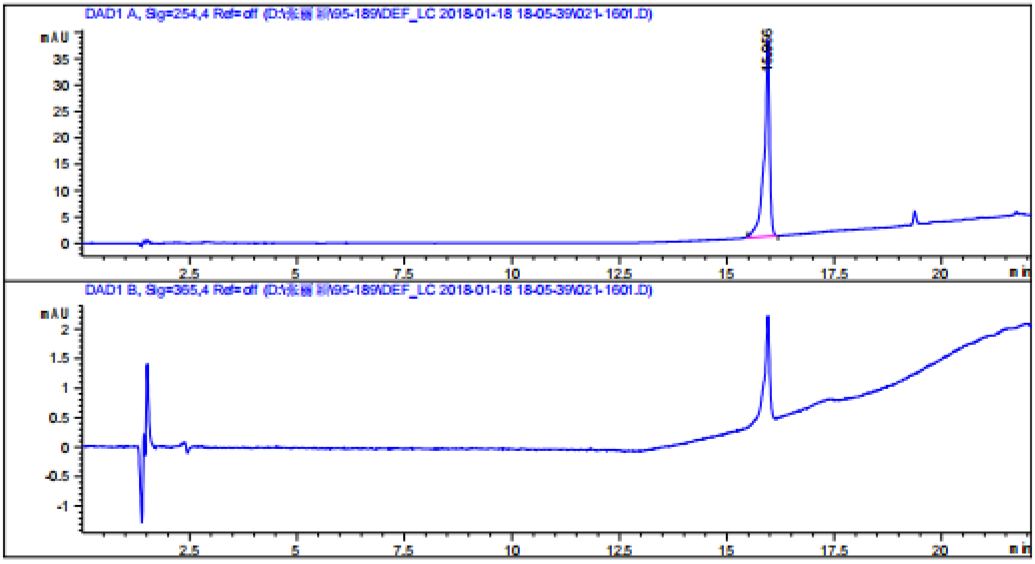

==
面积百分比报告
==

排序 : 信号
乘积因子: : 1.0000
稀释因子: : 1.0000
内标使用乘积因子和稀释因子

信号 1: DAD1 A, Sig=254,4 Ref=off

峰 #	保留时间 [min]	类型	峰宽 [min]	峰面积 [mAU*s]	峰高 [mAU]	峰面积 %
1	15.956	BB	0.1199	325.11526	37.21554	100.0000

总量 : 325.11526 37.21554

Figure B3. Purity of compound **4a**

（调用后修改）

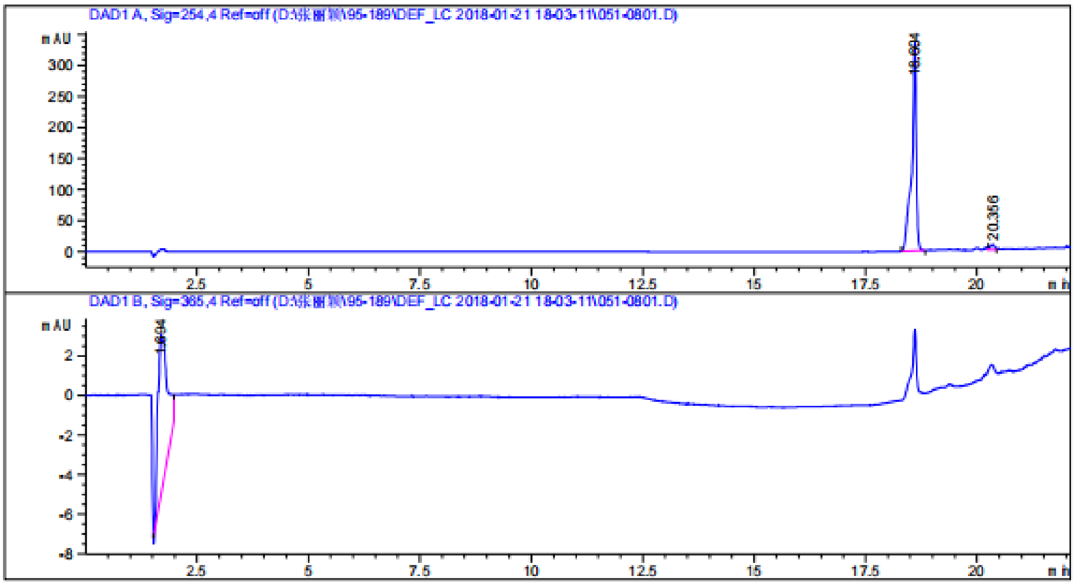

面积百分比报告

排序 : 信号
乘积因子: : 1.0000
稀释因子: : 1.0000
内标使用乘积因子和稀释因子

信号 1: DAD1 A, Sig=254,4 Ref=off

峰 #	保留时间 [min]	类型	峰宽 [min]	峰面积 [mAU*s]	峰高 [mAU]	峰面积 %
1	18.604	BB	0.0966	2323.89893	336.43353	97.4659
2	20.356	VV	0.0967	60.42156	8.73782	2.5341
总量 :				2384.32049	345.17135	

Figure B4. Purity of compound **5a**

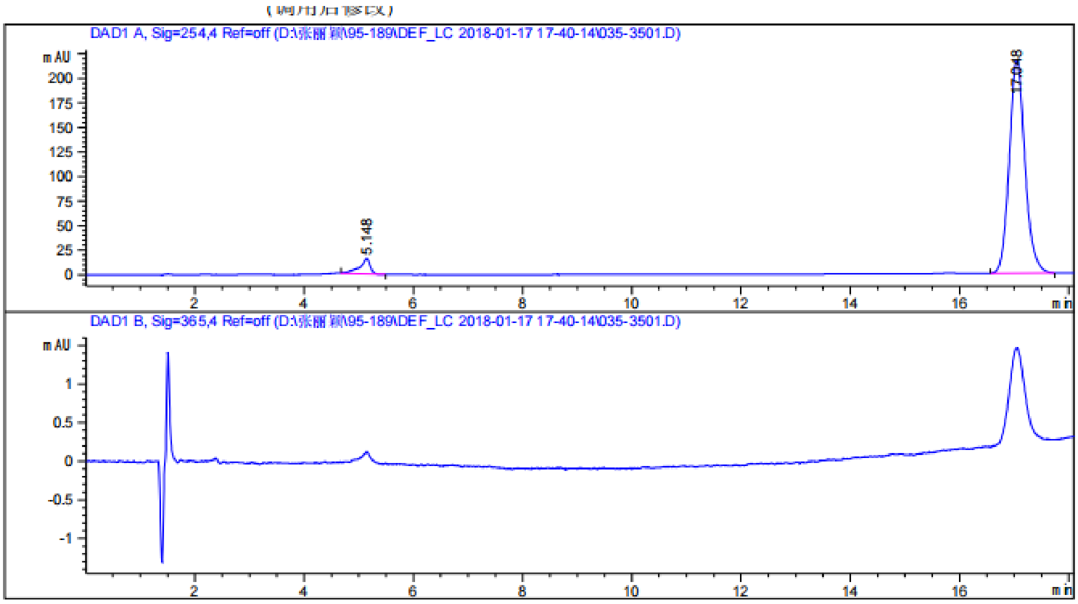

面积百分比报告

排序 : 信号
乘积因子: : 1.0000
稀释因子: : 1.0000
内标使用乘积因子和稀释因子

信号 1: DAD1 A, Sig=254,4 Ref=off

峰 #	保留时间 [min]	类型	峰宽 [min]	峰面积 [mAU*s]	峰高 [mAU]	峰面积 %
1	5.148	BB	0.1891	214.99921	15.76397	4.7199
2	17.048	BB	0.3055	4340.19434	216.89122	95.2801

总量 : 4555.19354 232.65519

Figure B5. Purity of compound **Ia**

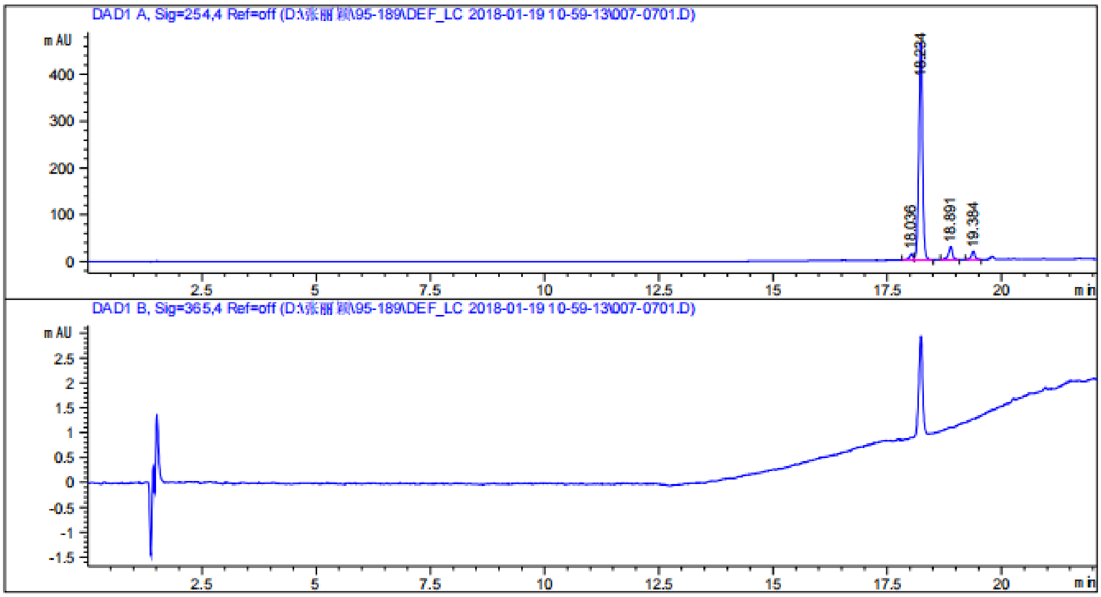

===

面积百分比报告

===

排序　　　　　　　　　:　　　信号
乘积因子:　　　　　　　　:　　1.0000
稀释因子:　　　　　　　　:　　1.0000
内标使用乘积因子和稀释因子

信号 1: DAD1 A, Sig=254,4 Ref=off

峰 #	保留时间 [min]	类型	峰宽 [min]	峰面积 [mAU*s]	峰高 [mAU]	峰面积 %
1	18.036	BV	0.0815	76.24767	13.18615	2.5665
2	18.234	VV	0.0840	2627.97803	464.59369	88.4595
3	18.891	VB	0.0851	167.85146	28.35583	5.6500
4	19.384	BB	0.0836	98.74887	17.57381	3.3240

总量 :　　2970.82602　523.70947

Figure B6. Purity of compound **3b**

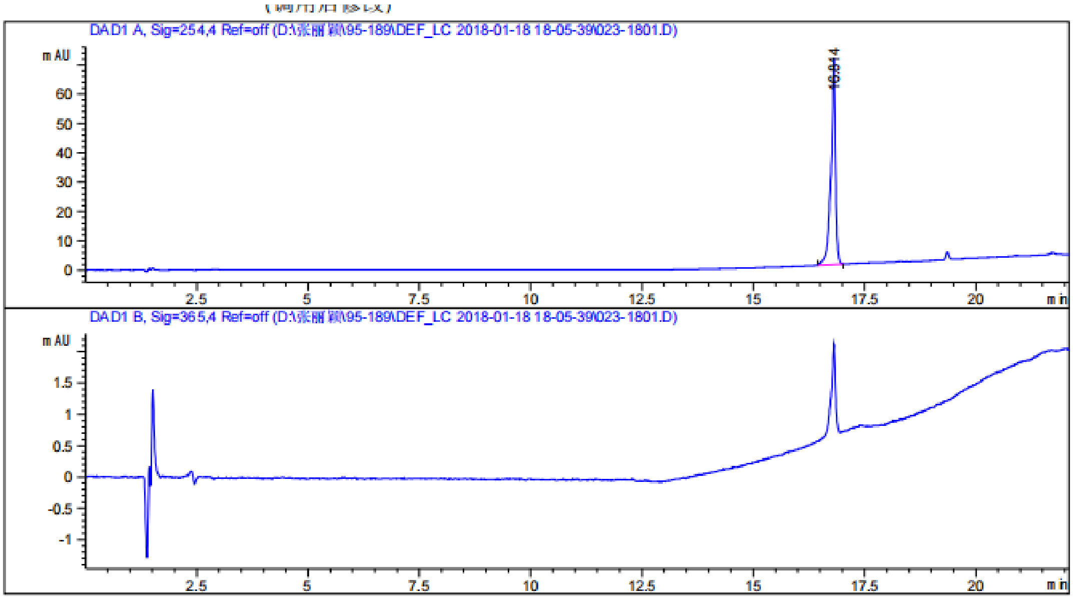

===

面积百分比报告

===

排序 : 信号
乘积因子: : 1.0000
稀释因子: : 1.0000
内标使用乘积因子和稀释因子

信号 1: DAD1 A, Sig=254,4 Ref=off

峰 #	保留时间 [min]	类型	峰宽 [min]	峰面积 [mAU*s]	峰高 [mAU]	峰面积 %
1	16.814	BB	0.1003	508.73264	70.44234	100.0000

总量 : 508.73264 70.44234

Figure B7. Purity of compound **4b**

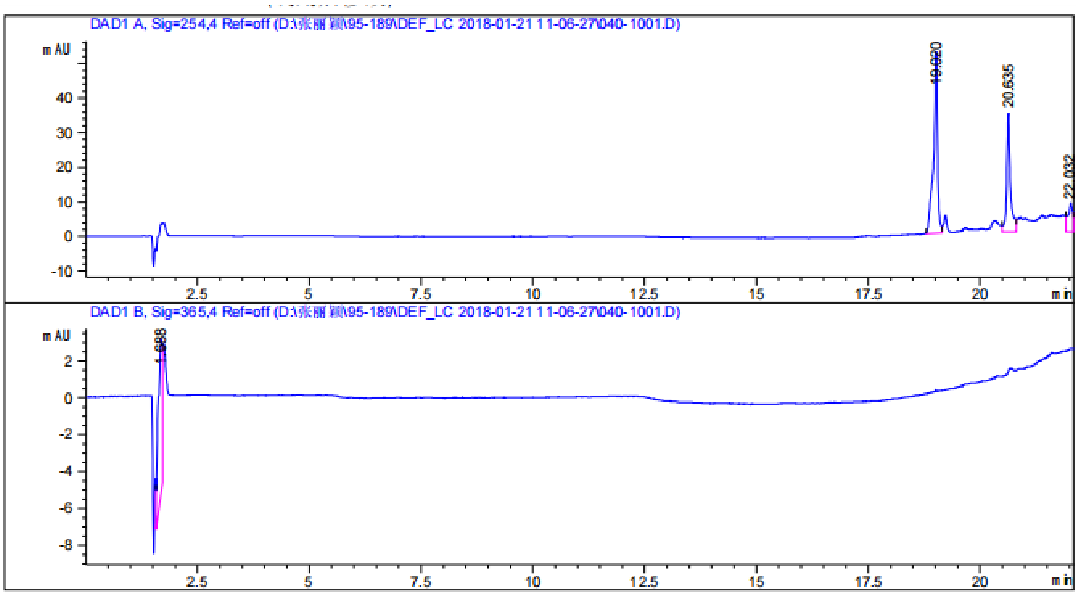

面积百分比报告

排序 : 信号
乘积因子: : 1.0000
稀释因子: : 1.0000
内标使用乘积因子和稀释因子

信号 1: DAD1 A, Sig=254,4 Ref=off

峰 #	保留时间 [min]	类型	峰宽 [min]	峰面积 [mAU*s]	峰高 [mAU]	峰面积 %
1	19.020	BV	0.0864	327.74884	52.79531	55.3674
2	20.635	VB	0.0846	202.54845	34.43367	34.2170
3	22.032	VBA	0.1009	61.65524	8.27552	10.4156

总量 : 591.95253 95.50451

Figure B8. Purity of compound **5b**

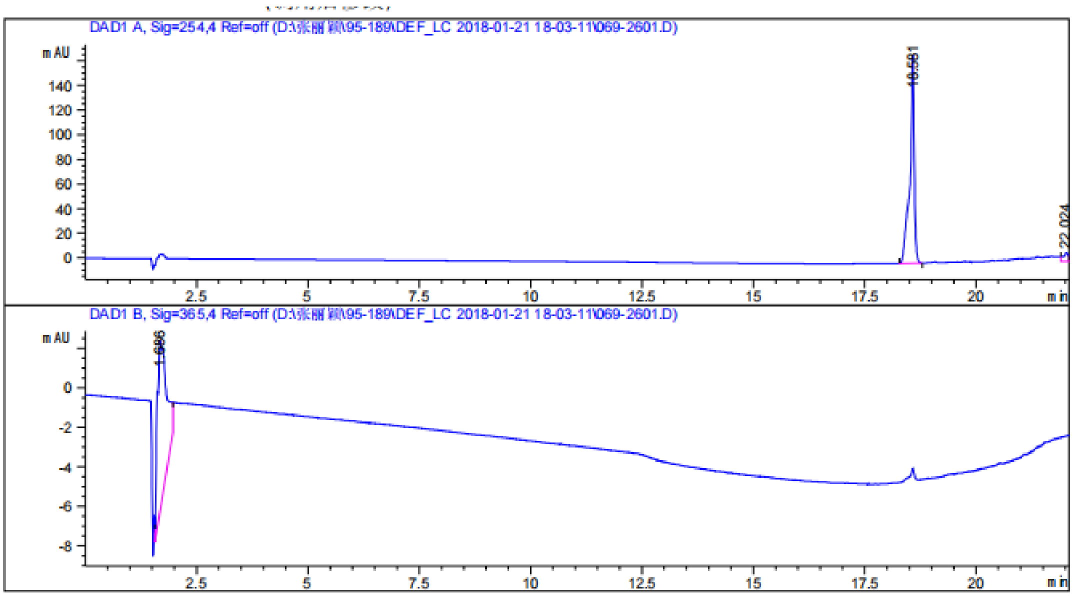

==

面积百分比报告

==

排序 : 信号
乘积因子: : 1.0000
稀释因子: : 1.0000
内标使用乘积因子和稀释因子

信号 1: DAD1 A, Sig=254,4 Ref=off

峰 #	保留时间 [min]	类型	峰宽 [min]	峰面积 [mAU*s]	峰高 [mAU]	峰面积 %
1	18.581	BB	0.0935	1151.45337	169.07153	95.0902
2	22.024	VBA	0.1004	59.45246	7.84970	4.9098

总量 : 1210.90583 176.92123

Figure B9. Purity of compound **Ib**

(调用后修改)

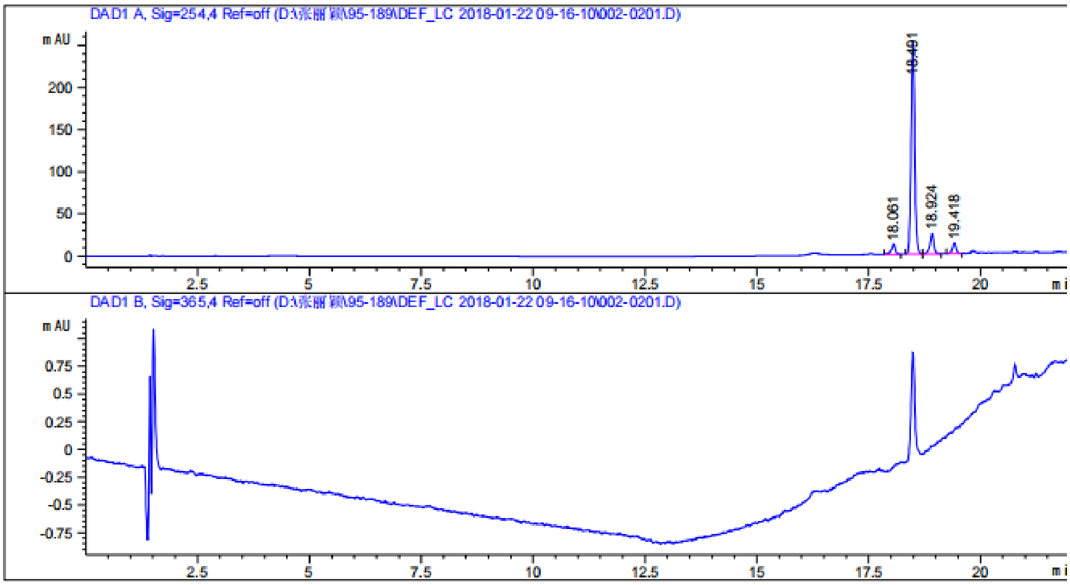

==

面积百分比报告

==

排序 : 信号
乘积因子: : 1.0000
稀释因子: : 1.0000
内标使用乘积因子和稀释因子

信号 1: DAD1 A, Sig=254,4 Ref=off

峰 #	保留时间 [min]	类型	峰宽 [min]	峰面积 [mAU*s]	峰高 [mAU]	峰面积 %
1	18.061	BB	0.0872	72.19878	11.83842	4.3222
2	18.491	BV	0.0818	1382.80579	252.84964	82.7824
3	18.924	VB	0.0851	141.81035	23.93137	8.4896
4	19.418	BB	0.0880	73.59492	12.62630	4.4058
总量 :				1670.40983	301.24574	

Figure B10. Purity of compound **3c**

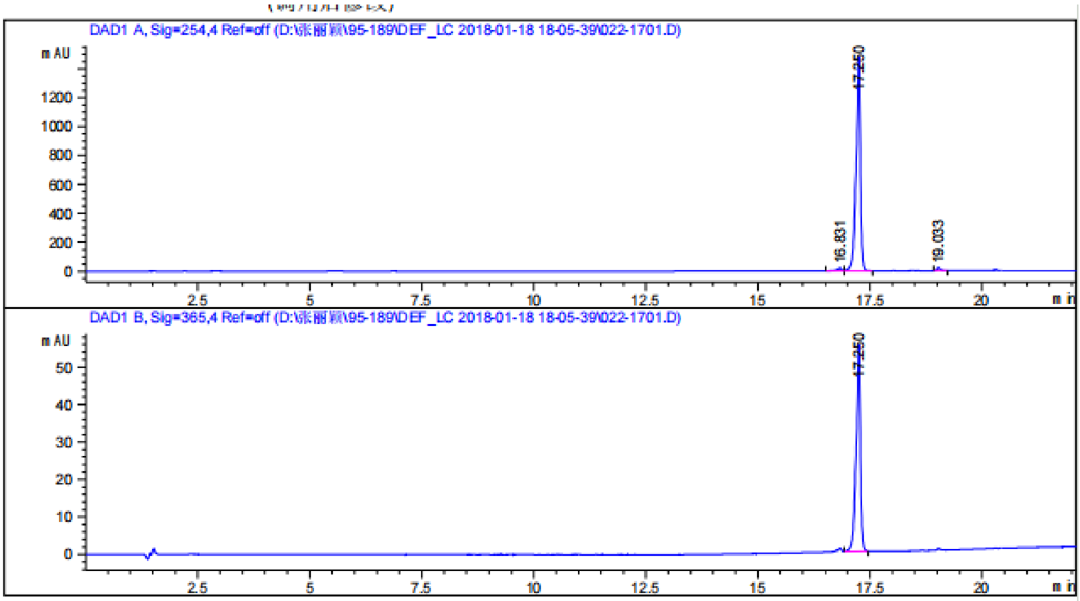

===

面积百分比报告

===

排序 : 信号
乘积因子: : 1.0000
稀释因子: : 1.0000
内标使用乘积因子和稀释因子

信号 1: DAD1 A, Sig=254,4 Ref=off

峰 #	保留时间 [min]	类型	峰宽 [min]	峰面积 [mAU*s]	峰高 [mAU]	峰面积 %
1	16.831	BV	0.1029	157.07838	21.09971	1.5327
2	17.250	VB	0.0945	9982.23730	1484.41235	97.4019
3	19.033	BB	0.0802	109.18395	20.46498	1.0654

总量 : 1.02485e4 1525.97704

Figure B11. Purity of compound **4c**

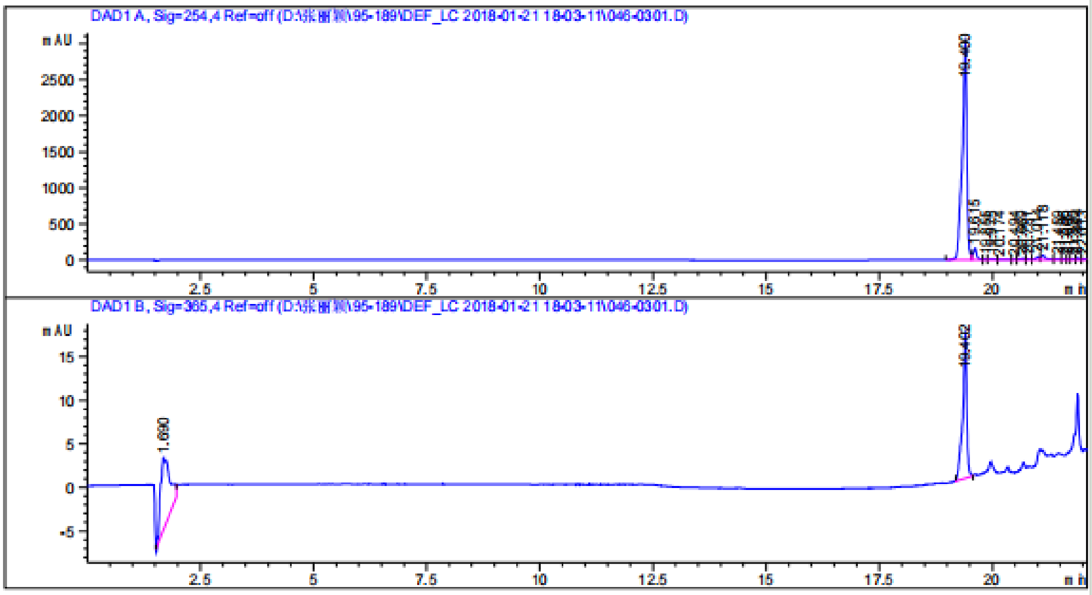

面积百分比报告

排序 : 信号
乘积因子: : 1.0000
稀释因子: : 1.0000
内标使用乘积因子和稀释因子

信号 1: DAD1 A, Sig=254,4 Ref=off

峰 #	保留时间 [min]	类型	峰宽 [min]	峰面积 [mAU*s]	峰高 [mAU]	峰面积 %
1	19.400	BV	0.1012	2.13590e4	2995.90771	87.0699
2	19.615	VV	0.0811	970.88318	168.84291	3.9578
3	19.855	VV	0.0897	82.51156	12.71784	0.3364
4	19.975	VV	0.1712	168.87238	14.36439	0.6884
5	20.174	VV	0.2047	167.53482	11.31975	0.6830
6	20.494	VV	0.0874	50.47502	8.02481	0.2058
7	20.669	VV	0.1190	168.98351	20.31652	0.6889
8	20.781	VV	0.0858	78.38342	12.38982	0.3195
9	21.014	VV	0.0878	297.66989	47.06881	1.2134
10	21.118	VV	0.0972	544.37024	76.35661	2.2191
11	21.459	VV	0.1320	139.40990	13.78265	0.5683
12	21.586	VV	0.0867	81.21391	13.03625	0.3311
13	21.669	VV	0.0706	61.77565	12.73072	0.2518
14	21.820	VV	0.0864	102.12440	15.98003	0.4163
15	21.884	VV	0.0829	148.62912	24.44403	0.6059
16	22.013	VBA	0.0929	109.02053	16.11084	0.4444

总量 : 2.45309e4 3463.39370

Figure B12. Purity of compound **5c**

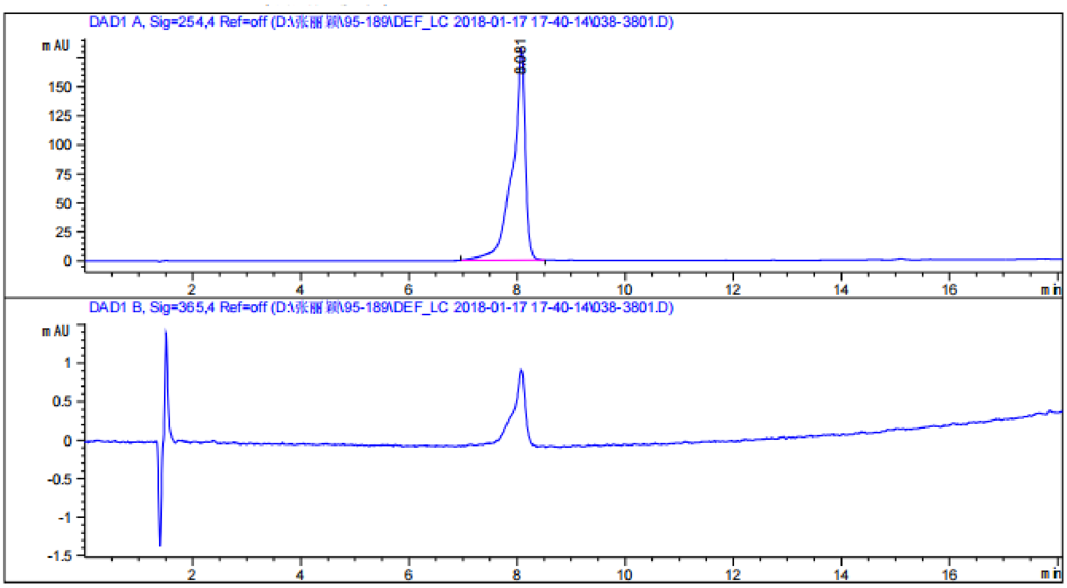

==

面积百分比报告

==

排序 : 信号
乘积因子: : 1.0000
稀释因子: : 1.0000
内标使用乘积因子和稀释因子

信号 1: DAD1 A, Sig=254,4 Ref=off

峰 #	保留时间 [min]	类型	峰宽 [min]	峰面积 [mAU*s]	峰高 [mAU]	峰面积 %
1	8.081	BB	0.2245	3006.43677	182.20821	100.0000

总量 : 3006.43677 182.20821

Figure B13. Purity of compound **Ic**

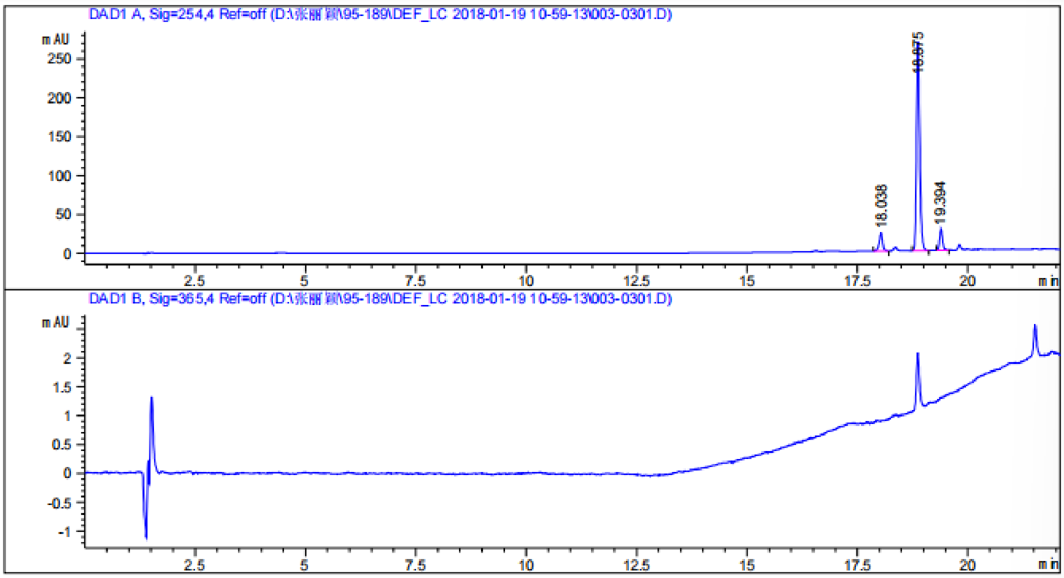

==

面积百分比报告

==

排序 : 信号
乘积因子: : 1.0000
稀释因子: : 1.0000
内标使用乘积因子和稀释因子

信号 1: DAD1 A, Sig=254,4 Ref=off

峰 #	保留时间 [min]	类型	峰宽 [min]	峰面积 [mAU*s]	峰高 [mAU]	峰面积 %
1	18.038	BB	0.0778	123.83688	23.36444	7.7019
2	18.875	BB	0.0768	1352.31152	268.47446	84.1051
3	19.394	BB	0.0714	131.73537	27.72933	8.1931

总量 : 1607.88377 319.56823

Figure B14. Purity of compound **2d**

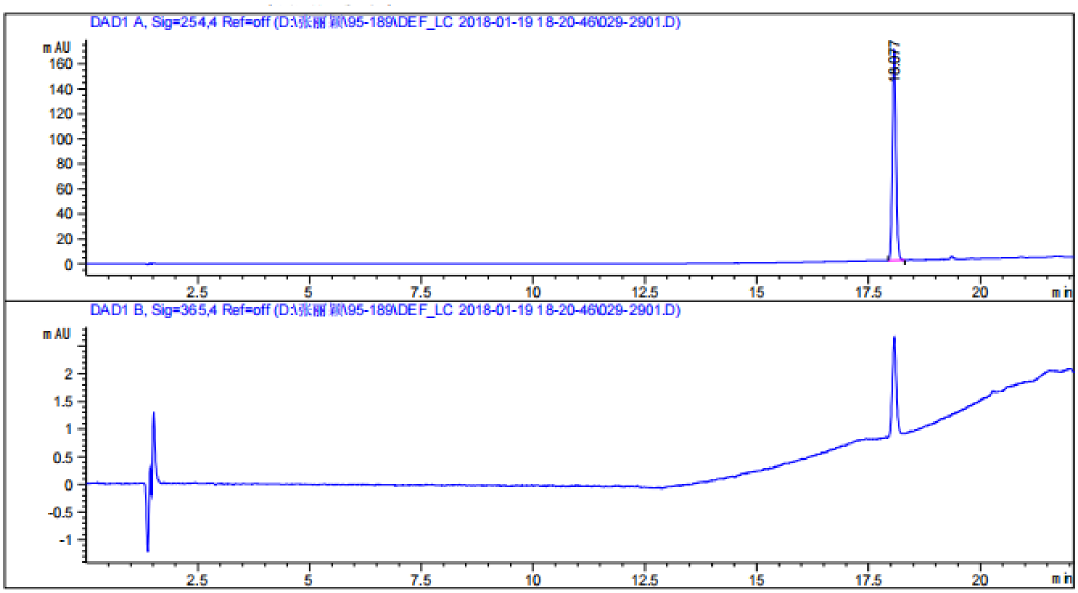

===

面积百分比报告

===

排序 : 信号
乘积因子: : 1.0000
稀释因子: : 1.0000
内标使用乘积因子和稀释因子

信号 1: DAD1 A, Sig=254,4 Ref=off

峰 #	保留时间 [min]	类型	峰宽 [min]	峰面积 [mAU*s]	峰高 [mAU]	峰面积 %
1	18.077	BB	0.0861	927.73950	168.80429	100.0000

总量 : 927.73950 168.80429

Figure B15. Purity of compound **3d**

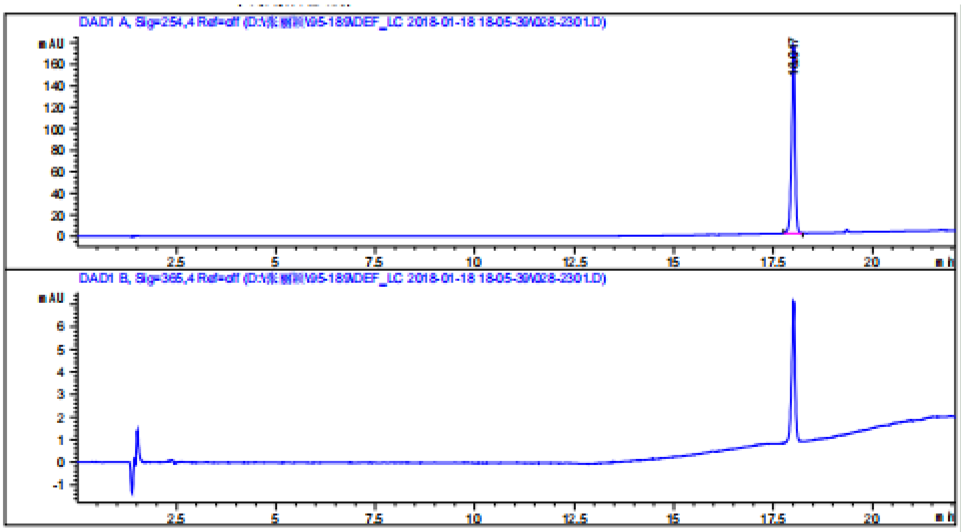

==

面积百分比报告

==

排序 : 信号
乘积因子: : 1.0000
稀释因子: : 1.0000
内标使用乘积因子和稀释因子

信号 1: DAD1 A, Sig=254,4 Ref=off

峰 #	保留时间 [min]	类型	峰宽 [min]	峰面积 [mAU*s]	峰高 [mAU]	峰面积 %
1	18.017	BB	0.0844	1024.95166	174.74547	100.0000

总量 : 1024.95166 174.74547

Figure B16. Purity of compound **4d**

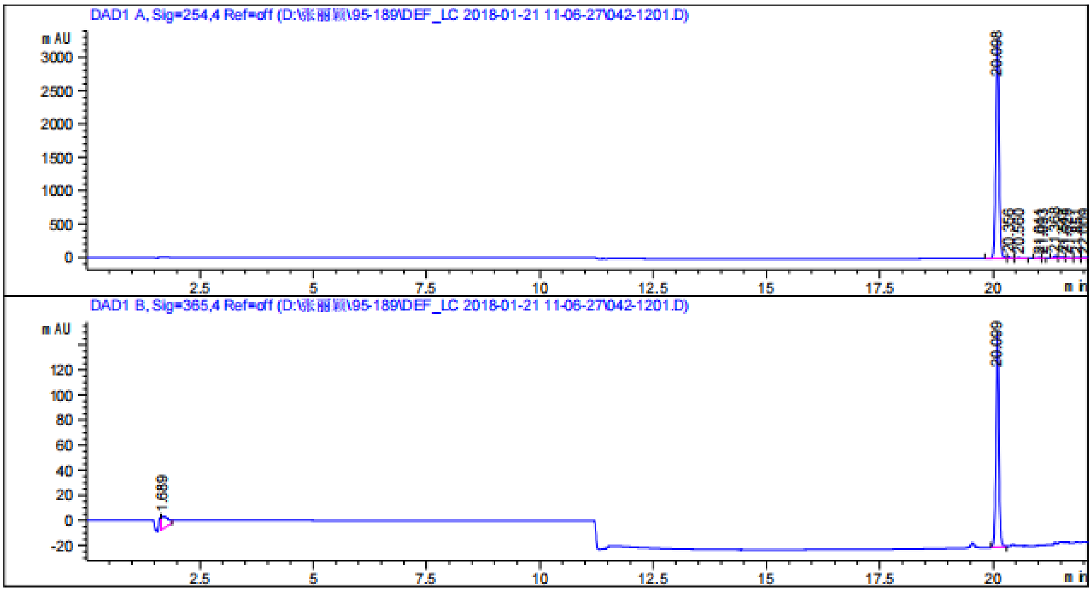

==

面积百分比报告

==

排序 : 信号
乘积因子: : 1.0000
稀释因子: : 1.0000
内标使用乘积因子和稀释因子

信号 1: DAD1 A, Sig=254,4 Ref=off

峰 #	保留时间 [min]	类型	峰宽 [min]	峰面积 [mAU*s]	峰高 [mAU]	峰面积 %
1	20.098	VV	0.0796	1.66860e4	3265.13525	92.6789
2	20.356	VV	0.0885	127.86318	20.02597	0.7102
3	20.560	VV	0.1572	167.78218	13.67229	0.9319
4	21.011	VV	0.1031	114.70485	16.10571	0.6371
5	21.093	VV	0.0651	56.46749	11.95976	0.3136
6	21.368	VV	0.0740	218.41161	42.44239	1.2131
7	21.543	VV	0.1167	194.70084	22.12785	1.0814
8	21.625	VV	0.1142	160.36415	19.05803	0.8907
9	21.851	VV	0.1247	138.01880	15.98477	0.7666
10	22.009	VBA	0.1065	139.77509	17.63266	0.7764

总量 : 1.80041e4 3444.14469

Figure B17. Purity of compound **5d**

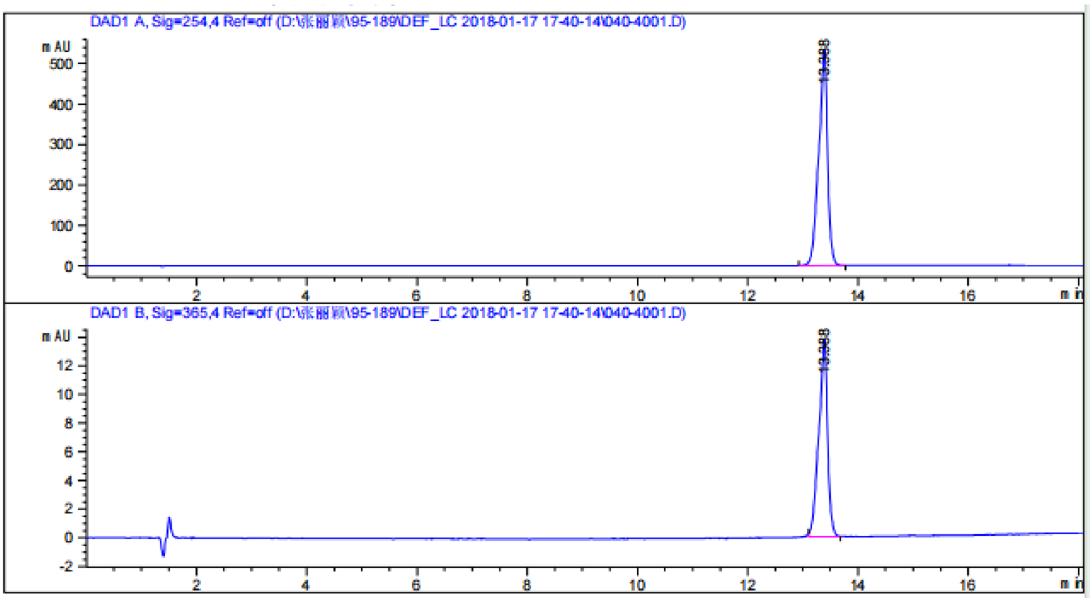

==

面积百分比报告

==

排序 : 信号
乘积因子: : 1.0000
稀释因子: : 1.0000
内标使用乘积因子和稀释因子

信号 1: DAD1 A, Sig=254,4 Ref=off

峰 #	保留时间 [min]	类型	峰宽 [min]	峰面积 [mAU*s]	峰高 [mAU]	峰面积 %
1	13.388	BB	0.1557	5931.74902	533.43445	100.0000
总量 :				5931.74902	533.43445	

Figure B18. Purity of compound **Id**

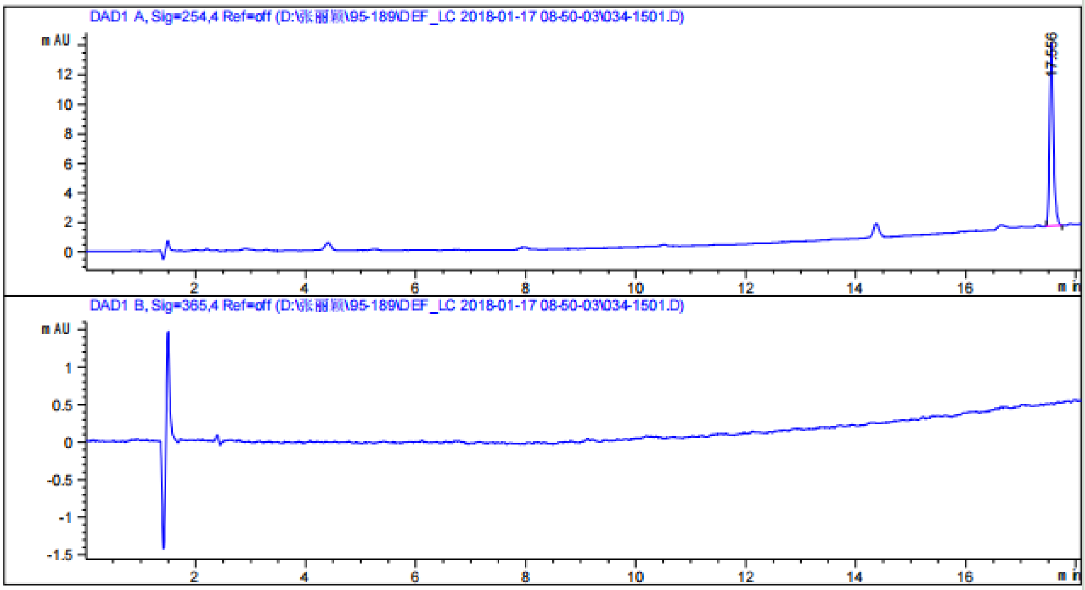

===

面积百分比报告

===

排序 : 信号
乘积因子: : 1.0000
稀释因子: : 1.0000
内标使用乘积因子和稀释因子

信号 1: DAD1 A, Sig=254,4 Ref=off

峰 #	保留时间 [min]	类型	峰宽 [min]	峰面积 [mAU*s]	峰高 [mAU]	峰面积 %
1	17.556	BB	0.0765	62.32247	12.42135	100.0000

总量 : 62.32247 12.42135

Figure B19. Purity of compound **3e**

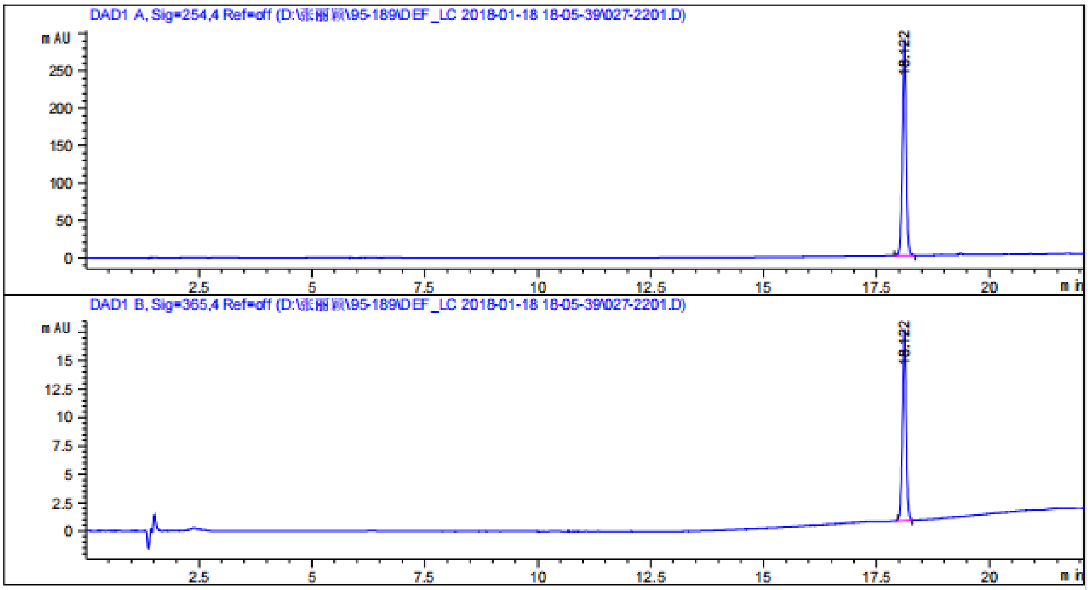

==
面积百分比报告
==

排序 : 信号
乘积因子: : 1.0000
稀释因子: : 1.0000
内标使用乘积因子和稀释因子

信号 1: DAD1 A, Sig=254,4 Ref=off

峰 #	保留时间 [min]	类型	峰宽 [min]	峰面积 [mAU*s]	峰高 [mAU]	峰面积 %
1	18.122	BB	0.0815	1613.08887	287.40308	100.0000

总量 : 1613.08887 287.40308

Figure B20. Purity of compound **4e**

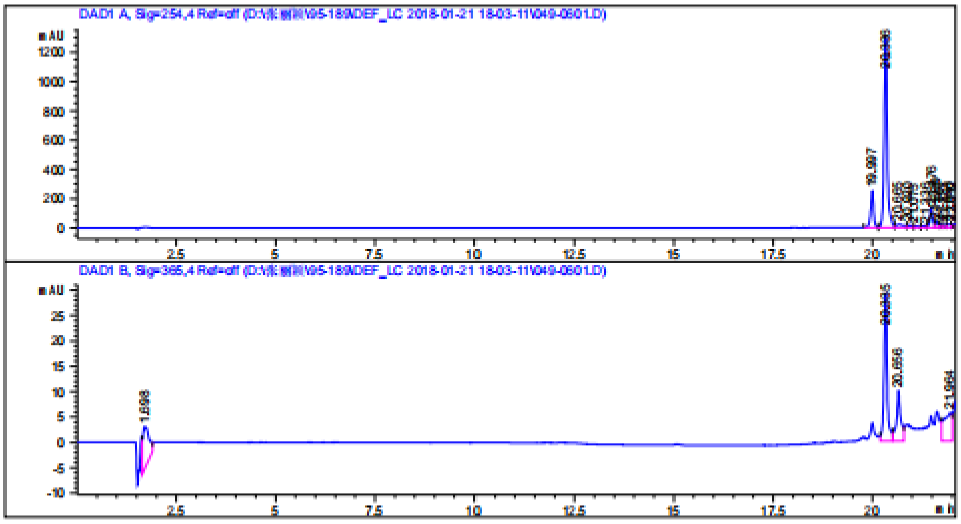

==

面积百分比报告

==

排序 : 信号
乘积因子: : 1.0000
稀释因子: : 1.0000
内标使用乘积因子和稀释因子

信号 1: DAD1 A, Sig=254,4 Ref=off

峰 #	保留时间 [min]	类型	峰宽 [min]	峰面积 [mAU*s]	峰高 [mAU]	峰面积 %
1	19.997	VV	0.0745	1301.71313	251.06619	12.5407
2	20.336	VV	0.0796	7079.18848	1299.08069	68.2010
3	20.665	VV	0.1759	313.61542	22.59772	3.0214
4	20.920	VV	0.1138	138.46385	16.51016	1.3340
5	21.075	VV	0.1464	109.05659	10.56452	1.0507
6	21.336	VV	0.0929	108.62357	15.67397	1.0465
7	21.476	VV	0.0688	652.79431	134.18454	6.2890
8	21.609	VV	0.0698	202.36789	42.27924	1.9496

峰 #	保留时间 [min]	类型	峰宽 [min]	峰面积 [mAU*s]	峰高 [mAU]	峰面积 %
9	21.707	VV	0.0614	93.01578	21.97711	0.8961
10	21.823	VV	0.0859	134.35213	21.19801	1.2944
11	21.940	VV	0.0909	160.24989	23.68435	1.54[illegible]8
12	22.012	VV	0.0539	86.44862	22.99918	0.8328

总量 : 1.03799e4 1881.81566

Figure B21. Purity of compound **5e**

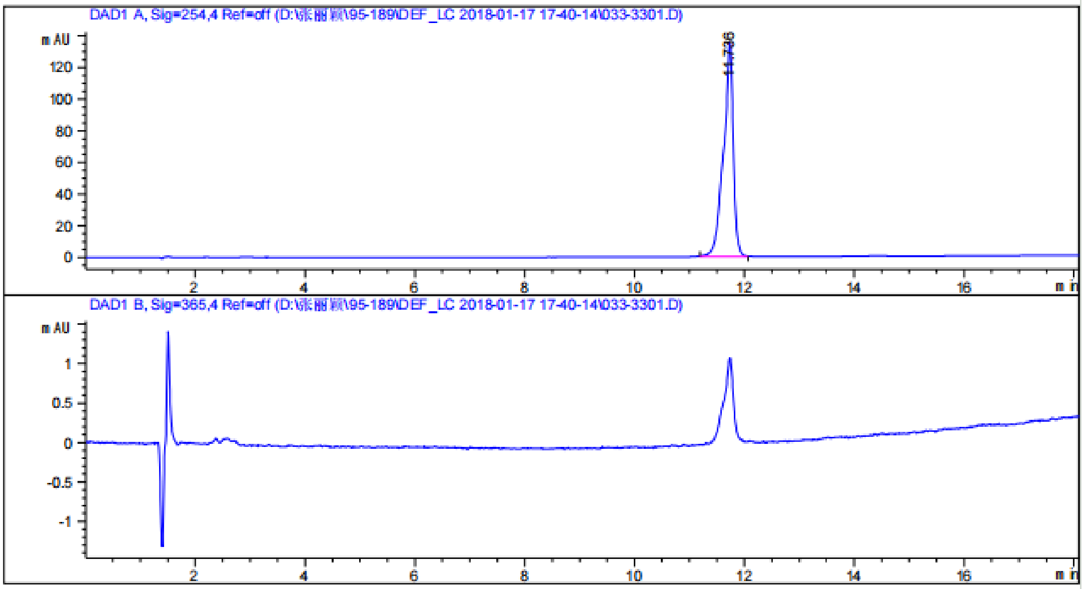

==

面积百分比报告

==

排序 : 信号

乘积因子 : 1.0000

稀释因子 : 1.0000

内标使用乘积因子和稀释因子

信号 1: DAD1 A, Sig=254,4 Ref=off

峰 #	保留时间 [min]	类型	峰宽 [min]	峰面积 [mAU*s]	峰高 [mAU]	峰面积 %
1	11.736	BB	0.1695	1663.77136	135.29851	100.0000

总量 : 1663.77136 135.29851

Figure B22. Purity of compound **3f**

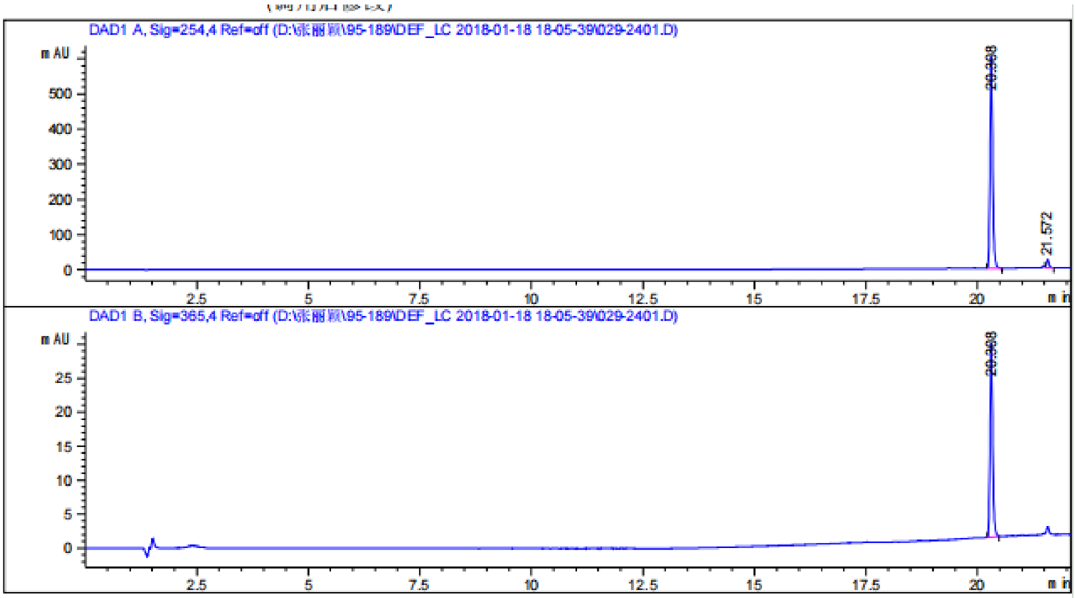

==

面积百分比报告

==

排序 : 信号
乘积因子: : 1.0000
稀释因子: : 1.0000
内标使用乘积因子和稀释因子

信号 1: DAD1 A, Sig=254,4 Ref=off

峰 #	保留时间 [min]	类型	峰宽 [min]	峰面积 [mAU*s]	峰高 [mAU]	峰面积 %
1	20.308	BB	0.0627	2517.88818	603.20740	96.4315
2	21.572	VB	0.0577	93.17590	23.76075	3.5685

总量 : 2611.06409 626.96814

Figure B23. Purity of compound **4f**

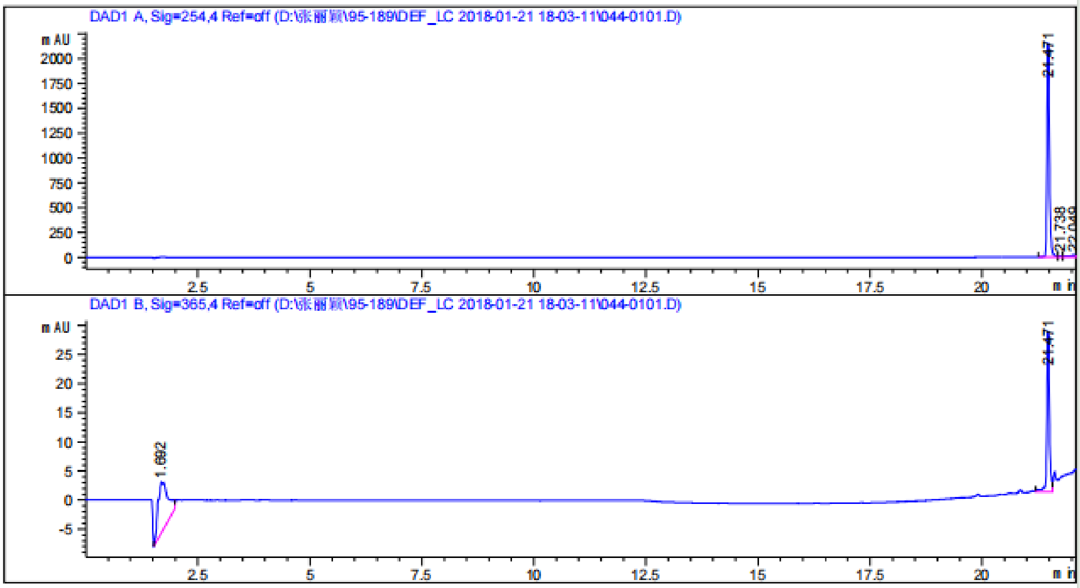

==
面积百分比报告
==

排序 : 信号
乘积因子: : 1.0000
稀释因子: : 1.0000
内标使用乘积因子和稀释因子

信号 1: DAD1 A, Sig=254,4 Ref=off

峰 #	保留时间 [min]	类型	峰宽 [min]	峰面积 [mAU*s]	峰高 [mAU]	峰面积 %
1	21.471	VV	0.0576	8045.80518	2154.15259	96.1792
2	21.738	VV	0.0900	83.48273	12.81357	0.9979
3	22.049	VBA	0.1669	236.14259	17.78609	2.8228

总量 : 8365.43050 2184.75224

Figure B24. Purity of compound **5f**

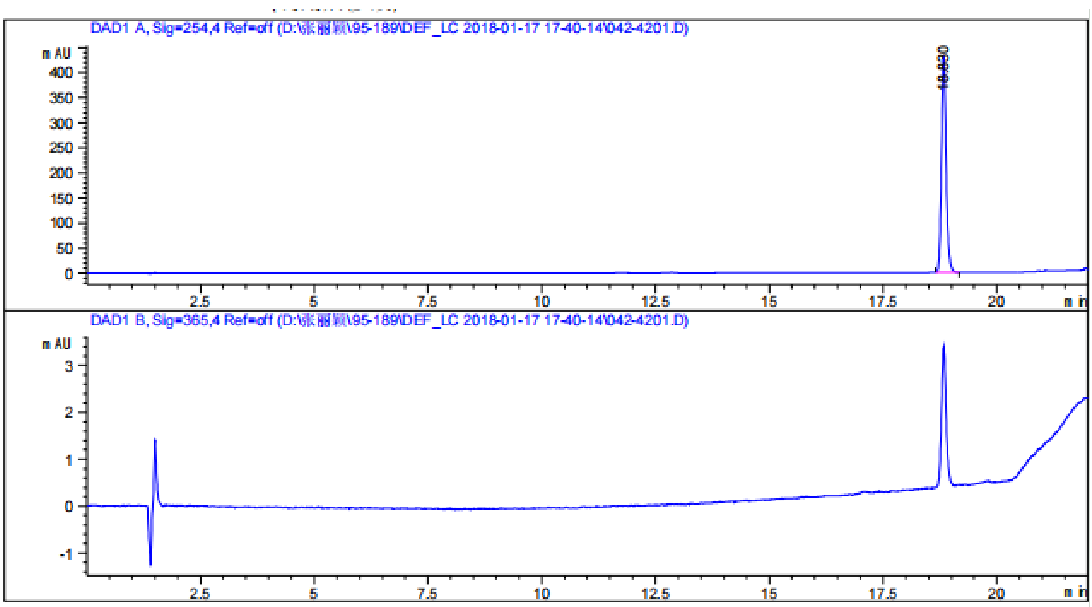

===

面积百分比报告

===

排序 : 信号
乘积因子: : 1.0000
稀释因子: : 1.0000
内标使用乘积因子和稀释因子

信号 1: DAD1 A, Sig=254,4 Ref=off

峰 #	保留时间 [min]	类型	峰宽 [min]	峰面积 [mAU*s]	峰高 [mAU]	峰面积 %
1	18.830	BB	0.1092	3074.96655	430.99625	100.0000

总量 : 3074.96655 430.99625

Figure B25. Purity of compound **If**

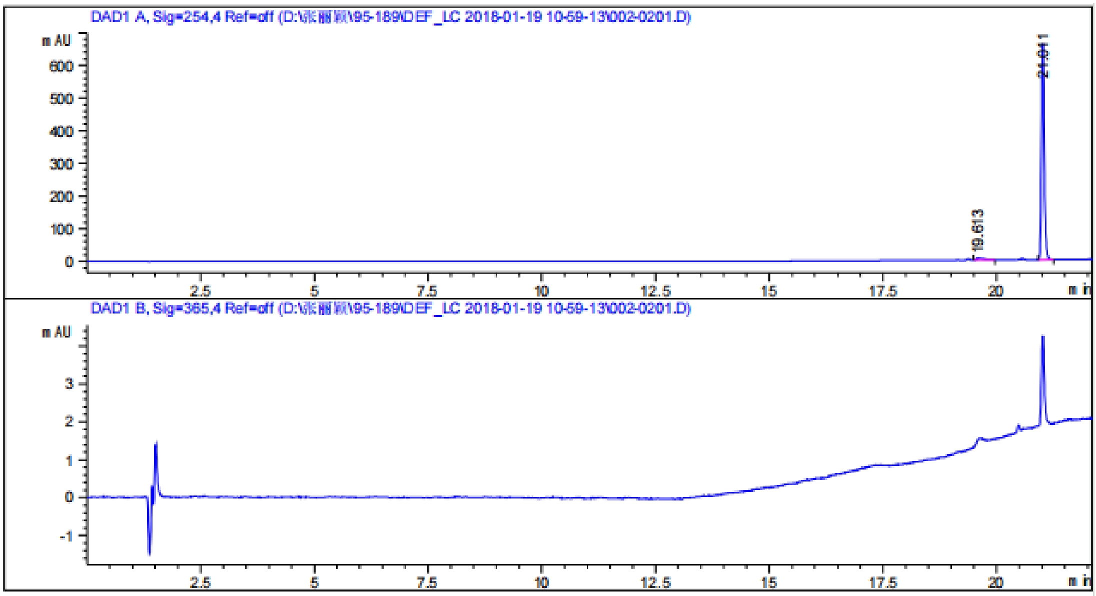

==

面积百分比报告

==

排序 : 信号
乘积因子: : 1.0000
稀释因子: : 1.0000
内标使用乘积因子和稀释因子

信号 1: DAD1 A, Sig=254,4 Ref=off

峰 #	保留时间 [min]	类型	峰宽 [min]	峰面积 [mAU*s]	峰高 [mAU]	峰面积 %
1	19.613	VB	0.1933	67.97404	5.53398	2.4148
2	21.011	BB	0.0623	2746.94189	664.05255	97.5852

总量 : 2814.91594 669.58653

Figure B26. Purity of compound **3g**

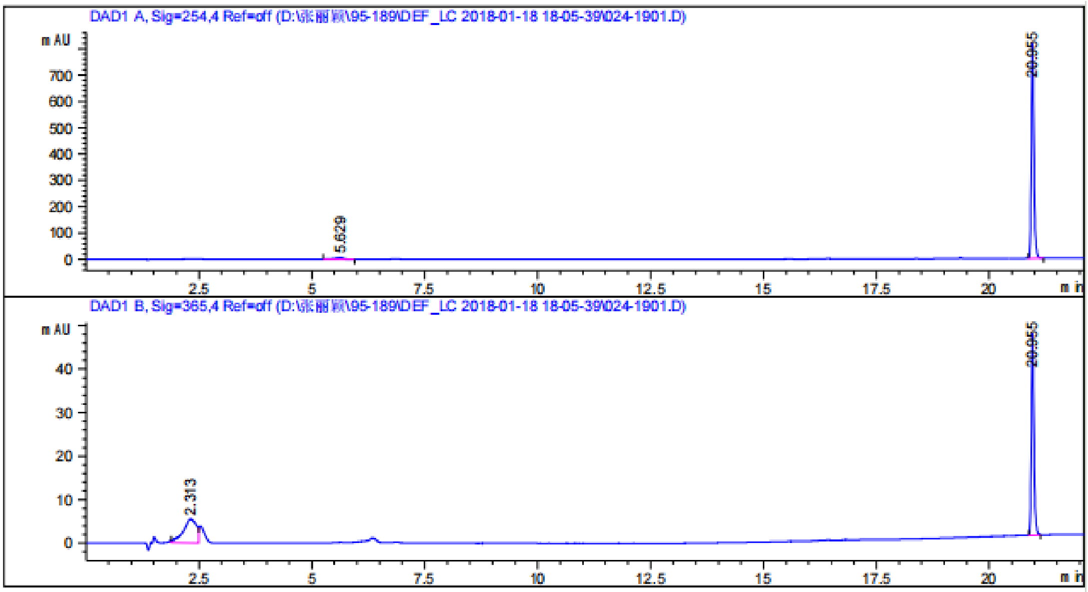

面积百分比报告

排序 : 信号
乘积因子: : 1.0000
稀释因子: : 1.0000
内标使用乘积因子和稀释因子

信号 1: DAD1 A, Sig=254,4 Ref=off

峰 #	保留时间 [min]	类型	峰宽 [min]	峰面积 [mAU*s]	峰高 [mAU]	峰面积 %
1	5.629	BB	0.1790	96.15900	7.52931	2.8982
2	20.955	BB	0.0600	3221.75488	816.31750	97.1018

总量 : 3317.91389 823.84681

Figure B27. Purity of compound **5g**

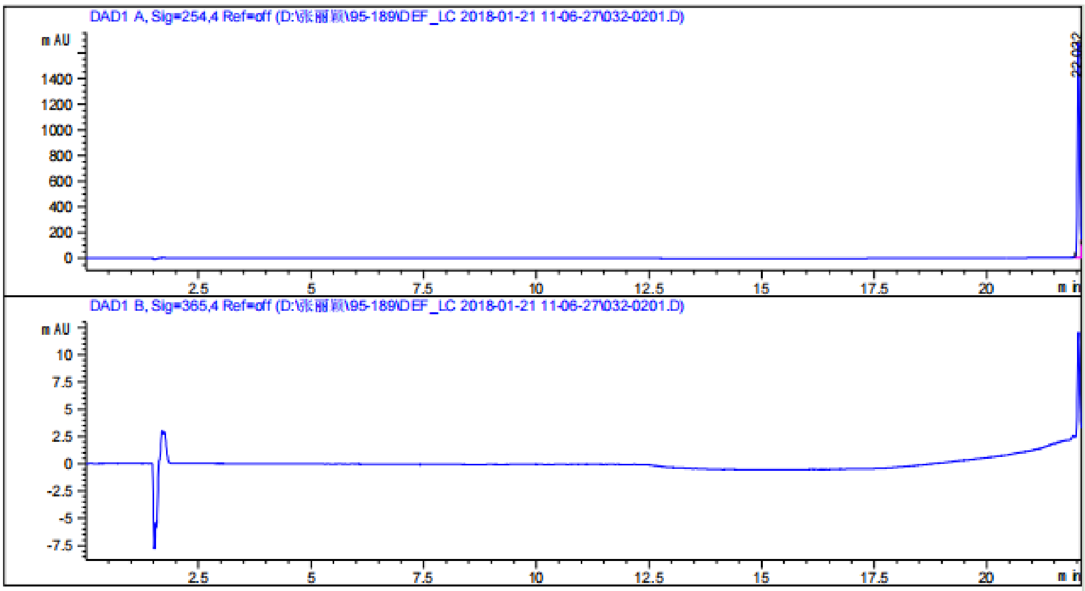

===

面积百分比报告

===

排序 : 信号
乘积因子: : 1.0000
稀释因子: : 1.0000
内标使用乘积因子和稀释因子

信号 1: DAD1 A, Sig=254,4 Ref=off

峰 #	保留时间 [min]	类型	峰宽 [min]	峰面积 [mAU*s]	峰高 [mAU]	峰面积 %
1	22.032	VBA	0.0508	5622.09961	1688.66492	100.0000

总量 : 5622.09961 1688.66492

Figure B28. Purity of compound **Ig**

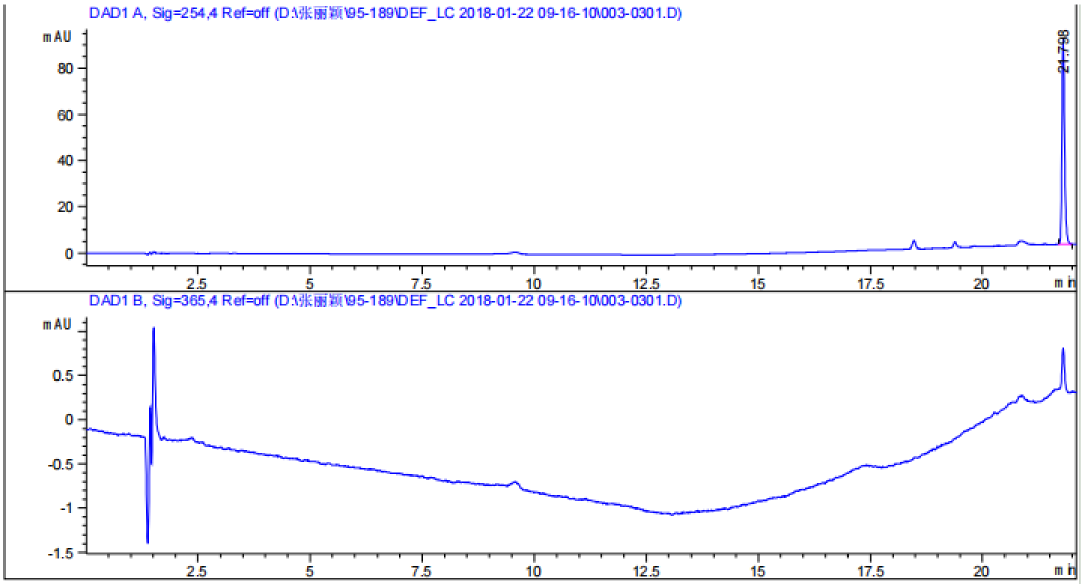

===

面积百分比报告

===

排序 : 信号

乘积因子: : 1.0000

稀释因子: : 1.0000

内标使用乘积因子和稀释因子

信号 1: DAD1 A, Sig=254,4 Ref=off

峰 #	保留时间 [min]	类型	峰宽 [min]	峰面积 [mAU*s]	峰高 [mAU]	峰面积 %
1	21.798	BB	0.0614	361.50043	88.97974	100.0000

总量 : 361.50043 88.97974

Figure B29. Purity of compound **IIa**

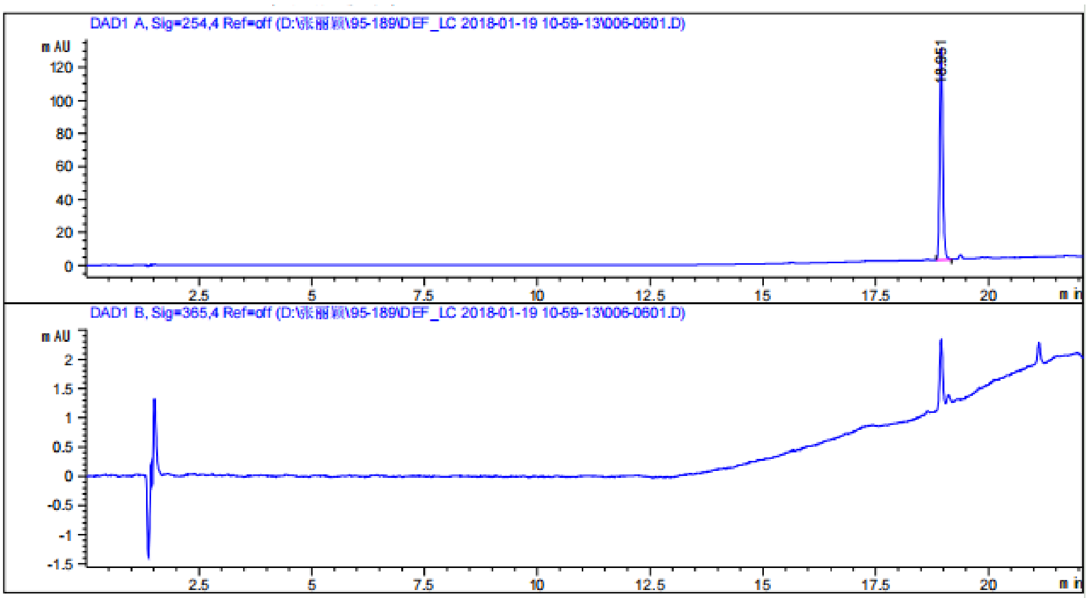

===

面积百分比报告

===

排序 : 信号
乘积因子 : 1.0000
稀释因子 : 1.0000
内标使用乘积因子和稀释因子

信号 1: DAD1 A, Sig=254,4 Ref=off

峰 #	保留时间 [min]	类型	峰宽 [min]	峰面积 [mAU*s]	峰高 [mAU]	峰面积 %
1	18.951	BB	0.0699	591.06219	127.93760	100.0000

总量 : 591.06219 127.93760

Figure B30. Purity of compound **IIc**

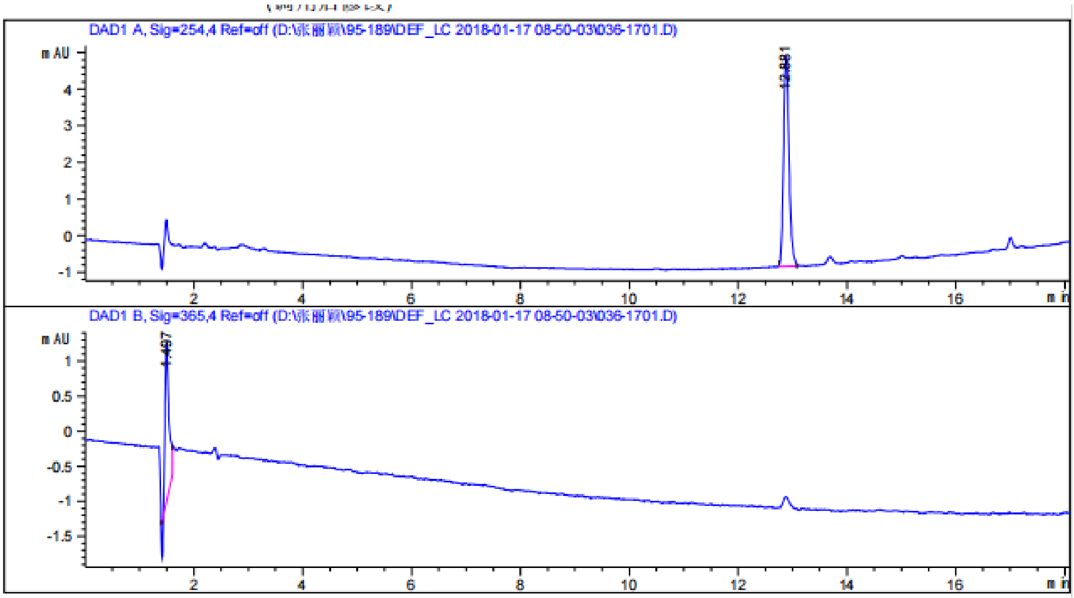

===

面积百分比报告

===

排序 : 信号
乘积因子: : 1.0000
稀释因子: : 1.0000
内标使用乘积因子和稀释因子

信号 1: DAD1 A, Sig=254,4 Ref=off

峰 #	保留时间 [min]	类型	峰宽 [min]	峰面积 [mAU*s]	峰高 [mAU]	峰面积 %
1	12.881	BB	0.1060	40.39581	5.74960	100.0000

总量 : 40.39581 5.74960

Figure B31. Purity of compound **IIe**

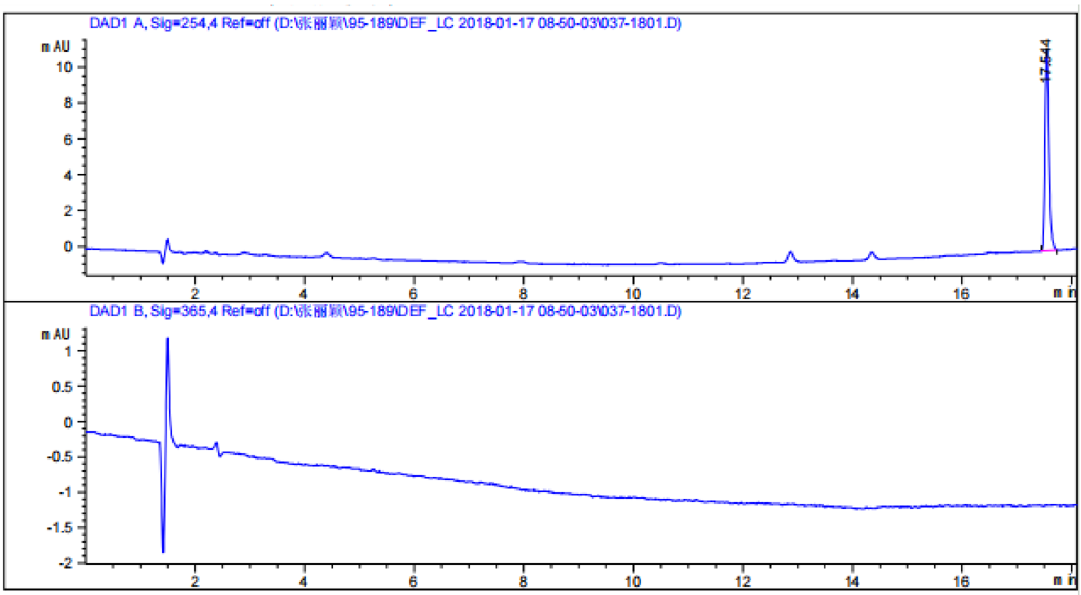

面积百分比报告

排序 : 信号
乘积因子 : 1.0000
稀释因子 : 1.0000
内标使用乘积因子和稀释因子

信号 1: DAD1 A, Sig=254,4 Ref=off

峰 #	保留时间 [min]	类型	峰宽 [min]	峰面积 [mAU*s]	峰高 [mAU]	峰面积 %
1	17.544	BB	0.0772	57.12691	11.25157	100.0000

总量 : 57.12691 11.25157

Figure B32. Purity of compound **IIf**

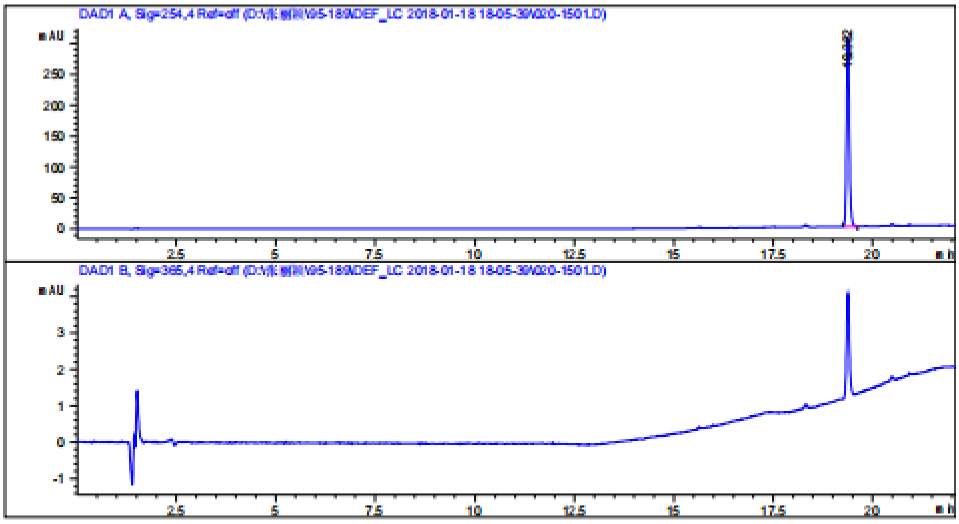

面积百分比报告

排序 : 信号
乘积因子: : 1.0000
稀释因子: : 1.0000
内标使用乘积因子和稀释因子

信号 1: DAD1 A, Sig=254,4 Ref=off

峰 #	保留时间 [min]	类型	峰宽 [min]	峰面积 [mAU*s]	峰高 [mAU]	峰面积 %
1	19.382	BB	0.0748	1485.97705	305.03360	100.0000

总量 : 1485.97705 305.03360
